# MicroRNA-33a-5p Modulates Japanese Encephalitis Virus Replication by Targeting Eukaryotic Translation Elongation Factor 1A1

**DOI:** 10.1128/JVI.03242-15

**Published:** 2016-03-11

**Authors:** Zheng Chen, Jing Ye, Usama Ashraf, Yunchuan Li, Siqi Wei, Shengfeng Wan, Ali Zohaib, Yunfeng Song, Huanchun Chen, Shengbo Cao

**Affiliations:** a State Key Laboratory of Agricultural Microbiology, Huazhong Agricultural University, Wuhan, Hubei, People's Republic of China; b Laboratory of Animal Virology, College of Veterinary Medicine, Huazhong Agricultural University, Wuhan, Hubei, People's Republic of China; c The Cooperative Innovation Center for Sustainable Pig Production, Huazhong Agricultural University, Wuhan, Hubei, People's Republic of China

## Abstract

Japanese encephalitis virus (JEV) is a typical mosquito-borne flavivirus responsible for acute encephalitis and meningitis in humans. However, the molecular mechanism for JEV pathogenesis is still unclear. MicroRNAs (miRNAs) are small noncoding RNAs that act as gene regulators. They are directly or indirectly involved in many cellular functions owing to their ability to target mRNAs for degradation or translational repression. However, how cellular miRNAs are regulated and their functions during JEV infection are largely unknown. In the present study, we found that JEV infection downregulated the expression of endogenous cellular miR-33a-5p. Notably, artificially transfecting with miR-33a-5p mimics led to a significant decrease in viral replication, suggesting that miR-33a-5p acts as a negative regulator of JEV replication. A dual-luciferase reporter assay identified eukaryotic translation elongation factor 1A1 (*EEF1A1*) as one of the miR-33a-5p target genes. Our study further demonstrated that *EEF1A1* can interact with the JEV proteins NS3 and NS5 in replicase complex. Through this interaction, *EEF1A1* can stabilize the components of viral replicase complex and thus facilitates viral replication during JEV infection. Taken together, these results suggest that miR-33a-5p is downregulated during JEV infection, which contributes to viral replication by increasing the intracellular level of *EEF1A1*, an interaction partner of JEV NS3 and NS5. This study provides a better understanding of the molecular mechanisms of JEV pathogenesis.

**IMPORTANCE** MiRNAs are critical regulators of gene expression that utilize sequence complementarity to bind to and modulate the stability or translation efficiency of target mRNAs. Accumulating data suggest that miRNAs regulate a wide variety of molecular mechanisms in the host cells during viral infections. JEV, a neurotropic flavivirus, is one of the major causes of acute encephalitis in humans worldwide. The roles of cellular miRNAs during JEV infections are widely unexplored. The present study explores a novel role of miR-33a-5p as a negative regulator of JEV replication. We found *EEF1A1* as a direct target of miR-33a-5p. We also demonstrated that *EEF1A1* interacts with and stabilize the components of JEV replicase complex, which positively regulates JEV replication. These findings suggest a new insight into the molecular mechanism of JEV pathogenesis and provide a possible therapeutic entry point for viral encephalitis.

## INTRODUCTION

The viral replication cycle requires the recruitment of specific host factors at various steps in the cycle. These host factors aid viral entry, genome replication, viral protein synthesis, and defense against host immune responses (1). A growing body of evidence has demonstrated that microRNAs (miRNAs) are one of the integral host factors that regulate viral replication and modulate host-virus interactions after infection.

miRNAs are small noncoding RNAs produced by hosts or viruses that regulate gene expression via base-pairing interactions with target mRNAs. They can regulate almost all biological processes, including cellular proliferation and differentiation, development, apoptosis, and host defense (2–6). Recent studies suggest that host miRNAs act in antiviral defense by regulating immune pathways during infection (7, 8). miRNAs can also act in host defense against invading viral pathogens by modulating the host cell environment or via direct targeting of the viral genome (9). Furthermore, accumulating evidence suggests a central role for host miRNAs in virus replication. For example, miR-382, miR-198, miR-223, miR-125b, and miR-28 inhibit HIV replication by modulating host cellular factors or by directly targeting the HIV genome (10, 11). Another host miRNA, miR-21, facilitates hepatitis C virus (HCV) replication by targeting host MyD88 and IRAK1 (12). Furthermore, miR-122 promotes HCV replication by enhancing its colony-forming ability (13). Similarly, influenza virus, human cytomegalovirus, and dengue virus regulate host miRNA expression profiles to facilitate their replication (14). Since the details of miRNA-mediated regulation of viral infection have only just begun to emerge, comprehensive investigation of their roles in viral pathogenesis will contribute to a better understanding of host-pathogen interactions.

Japanese encephalitis virus (JEV) belongs to the JEV serocomplex of the genus *Flavivirus* and family Flaviviridae (15, 16). It is a typical mosquito-borne flavivirus responsible for acute encephalitis and meningitis in humans (17). JEV is a single-stranded positive-sense RNA virus consisting of three structural proteins, namely, envelope (E), capsid (C), and premembrane (PrM), and seven nonstructural (NS) proteins, NS1, NS2A, NS2B, NS3, NS4A, NS4B, and NS5 (18). After transfer to the host via the bite of an infected mosquito, JEV infects the lymph nodes and begins to replicate. Flavivirus replication begins with RNA-dependent RNA polymerization by a viral replicase complex (19, 20), of which NS3 and NS5 are major components promoting efficient viral replication in close association with host factors (19). It is reported that hnRNP A2 can interact with JEV NS5 and core protein to regulate viral replication (21). Our previous study found that HSP70 can interact with JEV NS5 and NS3 and facility viral replication (20). These prompt that host factors play an important role in JEV replication process.

Since the roles of host miRNAs in JEV replication has rarely been reported, we have a strong interest in exploring how miRNAs participate in JEV replication. Here, we examined the role of cellular miR-33a-5p on JEV infection. We found that miR-33a-5p negatively regulates JEV replication by targeting eukaryotic translation elongation factor 1A1 (*EEF1A1*), thus clarifying one of the molecular mechanisms underlying JEV pathogenesis.

## MATERIALS AND METHODS

### Cells and viruses.

The human embryonic kidney (HEK293T) and baby hamster kidney (BHK-21) cell lines were cultured in Dulbecco modified Eagle medium (DMEM; Sigma) supplemented with 100 U/ml penicillin, 100 μg/ml streptomycin, and 10% fetal bovine serum (Gibco) at 37°C and 5% CO_2_. The JEV P3 strain used in this study was generated by our laboratory.

### miR-33a-5p mimics and inhibitors.

Human miR-33a-5p mimics, inhibitors, and their controls were purchased from GenePharma. The sequences of the mimics, inhibitors, or scrambled oligonucleotides were as follows: miR-33a-5p mimics, 5′-GUGCAUUGUAGUUGCAUUGCA-3′ (forward) and 5′-CAAUGCAACUACAAUGCACUU-3′ (reverse); mimic negative controls, 5′-UUCUCCGAACGUGUCACGUTT-3′ (forward) and 5′-ACGUGACACGUUCGGAGAATT-3′ (reverse); miR-33a-5p inhibitor, 5′-UGCAAUGCAACUACAAUGCAC-3′; and inhibitor negative control, 5′-CAGUACUUUUGUGUAGUACAA-3′.

### Plasmid construction.

To construct wild-type psiCheck-2-*EEF1A1* 3′ untranslated region (UTR), the 3′ UTR of *EEF1A1* was amplified from cDNA derived from HEK293T cells. The PCR product was digested with PmeI and XhoI and cloned into the psiCheck-2 luciferase reporter vector. The cDNA of human *EEF1A1* was amplified by PCR and cloned into pCMV-Tag1 with the Myc tag fused at the 3′ end of the insert sequence. All plasmids were verified by DNA sequencing.

### Antibodies.

Mouse monoclonal antibodies against JEV NS3 and NS5 were generated in our laboratory. Commercially obtained antibodies used were: rabbit polyclonal anti-*EEF1A1* (ABclonal Technology), mouse monoclonal anti-Flag (ABclonal Technology), mouse monoclonal anti-Myc (Abcam), mouse monoclonal anti-GAPDH (ABclonal Technology), mouse monoclonal anti-dsRNA J2 (English & Scientific Consulting Bt), horseradish peroxidase-conjugated anti-mouse and anti-rabbit IgG secondary antibodies (Boster), and Alexa Fluor 488-conjugated goat anti-mouse IgG and Alexa Fluor 555-conjugated goat anti-rabbit IgG (Invitrogen).

### Transfection of cells with miRNA mimics/inhibitors and viral infection.

All of the miRNA mimics (50 nmol/well) or inhibitors (100 nmol/well) were transfected into HEK293T cells (10^6^/well) in 12-well plates using Lipofectamine 2000 (Invitrogen). For the viral infection experiments, cells were infected with JEV P3 at the indicated multiplicity of infection (MOI) at 24 h posttransfection. Cells or supernatants from cell cultures were collected 24 h postinfection.

### Dual-luciferase reporter assay.

For the *EEF1A1* 3′-UTR luciferase reporter assay, HEK293T cells were cotransfected with 300 ng of the psiCheck-2 dual-luciferase plasmid described above, along with 25 nM miR-33a-5p mimics or mimic negative controls. After 24 h of incubation, firefly and Renilla luciferase activities were measured using dual-luciferase reporter assay system (Promega). The data are expressed as the firefly luciferase activity relative to the Renilla luciferase activity and are representative of three independent experiments.

### RNA extraction and quantitative real-time PCR.

Total RNA in treated cells was extracted using TRIzol Reagent (Invitrogen), and 1 μg of RNA was used to synthesize cDNA using a first-strand cDNA synthesis kit (Toyobo). Quantitative real-time PCR was performed using a 7500 real-time PCR system (Applied Biosystems) and SYBR green PCR master mix (Toyobo). Data were normalized to the level of β-actin expression in each sample. To quantify mature miRNA expression, a commercial Bulge-Loop miRNA quantitative reverse transcription-PCR (RT-PCR) detection method was used. Briefly, 1 μg total RNA was used as the template and reverse transcribed using a miR-33a-5p-specific RT primer. The resulting cDNA was used for quantitative real-time PCR with a universal reverse primer and a specific forward primer. Amplification was performed for 2 min at 50°C and 10 min at 95°C, followed by 40 cycles of 95°C for 15 s, 60°C for 15 s, and 72°C for 30 s. The relative expression of miRNAs was normalized to that of internal control U6 small nuclear RNA within each sample using the 2^−ΔΔ*CT*^ method. Expression was then standardized to the miRNA levels in mock-infected or control miRNA-treated cells. Primers used are listed in Table 1.

**TABLE 1:** Primers used in this study

Primer	Sequence (5′–3′)
*EEF1A1* F	GCGGATCCATGGGAAAGGAAAAGACT
*EEF1A1* R	GCCTCGAG TTTAGCCTTCTGAGCTTT
*EEF1A1*-UTR F	CGCTCGAGTTCTCAGACTATCCACCTT
*EEF1A1*-UTR R	CGGTTTAAACAAACAACCCTATTCTCCAC
*EEF1A1*-UTR-Mut R	TTTACGATCGAATCTTATCAT
*EEF1A1*-UTR-Mut F	ATGATAAGATTCGATCGTAAA
hsa-miR-33a-5p F	GTGCATTGTAGTTGCATTGCA
hsa-miR-33a-5p R	GTGCAGGGTCCGAGGT
hsa-miR-33a-5p-loop F	GTCGTATCCAGTGCAGGGTCCGAGGTATTCGCACTGGATACGACTGCAAT
Universal reverse primer	GTGCAGGGTCCGAGGT
pri-miR-33a-5p F	GGCAGCCTTGGAGTGGGTTC
pri-miR-33a-5p R	GCTGCCCGCCAGGAGGTATG
pre-miR-33a-5p F	TGCATGTTCTGGTGGTAC
pre-miR-33a-5p R	TGTGATGCACTGTGGAAA

### Western blotting.

Total cellular lysates were prepared using radioimmunoprecipitation assay buffer (Sigma) containing protease inhibitors (Roche). After sonication, protein concentrations were determined using a BCA protein assay kit (Thermo Scientific). Equal protein quantities were separated by SDS-PAGE and transferred to a polyvinylidene fluoride membrane (Millipore) using a Mini Trans-Blot Cell (Bio-Rad). Blots were probed with the relevant antibodies, and proteins were detected using enhanced chemiluminescence reagent (Thermo Scientific).

### Coimmunoprecipitation analysis.

Coimmunoprecipitation was performed according to our previous study (20). HEK293T cells (10^7^) were transfected with the indicated plasmids or were infected with JEV P3 at an MOI of 1.0. At 48 h posttransfection or postinfection, the cells were harvested and lysed with radioimmunoprecipitation assay buffer (Sigma) containing protease inhibitor cocktail (Roche). Each cell lysate was incubated with the relevant antibodies at 4°C overnight with gentle shaking. Protein A+G-agarose beads (25 μl; Beyotime) were added, and the samples were incubated for another 3 h. The agarose beads were subsequently washed three or more times with wash buffer (0.05 M Tris-HCl with 0.15 M NaCl). The bound proteins were eluted by boiling with SDS-PAGE loading buffer for 5 min and then subjected to Western blotting.

### Immunofluorescence analysis.

HEK293T cells were transfected with the *EEF1A1*-Myc plasmid, followed by infection with JEV P3 at an MOI of 1.0. At 36 h postinfection, the cells were fixed with ice-cold methanol for 10 min and then washed with phosphate-buffered saline, followed by incubation with the appropriate primary antibodies for 1 h at room temperature. After washing, the cells were incubated with a 1:500 dilution of Alexa Fluor 488- and Alexa Fluor 555-conjugated secondary antibodies for 30 min and then stained with DAPI (4′,6′-diamidino-2-phenylindole; Invitrogen) for another 10 min. The cells were finally washed and observed using a confocal microscope (Zeiss) with ×1,000 magnification.

### RNA interference.

The short hairpin RNA (shRNA) corresponding to the *EEF1A1* mRNA sequence (5′-CCTTGGTTCAAGGGATGGAAA-3′; sh-*EEF1A1*), which was used to inhibit endogenous *EEF1A1* protein expression, and the negative-control shRNA (sh-NC), which exhibited no downregulation of any human genes, were purchased from GeneCopoeia. HEK293T cells at 80% confluence were transfected with 2 μg of shRNA plasmid/well in 12-well plates using the X-tremeGENE HP DNA transfection reagent (Roche).

### Plaque assay.

Cells were first transfected with the indicated plasmids for 24 h and then infected with JEV at an MOI of 1.0. At 12, 24, and 36 h postinfection, cell suspensions were harvested, serially diluted, and then used to inoculate monolayers of BHK-21 cells. After 1 to 2 h of absorption, the cells were washed with serum-free DMEM and cultured for 3 to 5 days in DMEM containing 3% fetal bovine serum and 1.5% sodium carboxymethyl cellulose (Sigma). The cells were then stained with crystal violet for 2 h, followed by fixation with 10% formaldehyde overnight. Visible plaques were counted, and the viral titers were calculated. All data are expressed as means of triplicate samples.

### IP-RT-PCR.

Immunoprecipitation–RT-PCR (IP-RT-PCR) was performed as described previously (20). Cells (10^7^) transfected with plasmid *EEF1A1*-Myc were infected with JEV at an MOI of 1.0. At 36 h postinfection, the cells were harvested with RNA-protein binding buffer (10 mM HEPES [pH 7.3], 500 mM KCl, 1 mM EDTA, 2 mM MgCl_2_, 0.1% NP-40, 0.1 μg/μl yeast tRNA, 1 U/ml RNase inhibitor [Toyobo], and protease inhibitor cocktail [Roche]). After centrifugation at 13,000 × *g* at 4°C for 10 min, the supernatants were incubated with 20 μl of protein A+G-agarose beads and 2 μg of normal mouse IgG for 2 h at 4°C. After centrifugation, the supernatants were further incubated with anti-Myc at 4°C overnight; 25 μl of protein A+G-agarose beads was then added, and the samples were incubated for another 3 h. The agarose beads were subsequently washed three times with RNA-protein binding buffer lacking yeast tRNA, and RNA was isolated with TRIzol reagent. RT-PCR was carried out using the ReverTra Ace qPCR RT kit (Toyobo), followed by PCR with LA *Taq* polymerase (TaKaRa) and the primer set (5′-GGGGGCGTGTTTTGGGA-3′/5′-TCTCTTCCCTGCTGCAAA-3′) targeting the gene encoding JEV NS3.

### Ubiquitination analysis.

To measure ubiquitination level of NS3 and NS5 *in vitro*, HEK293T cells were transfected with *EEF1A1* shRNA or control shRNA or transfected with miR-33a-5p mimics or mimic negative controls. At 24 h posttransfection, the cells were infected with JEV. MG132 (20 nM) was added during the last 12 h of the culture. The cells were harvested and lysed with radioimmunoprecipitation assay buffer containing 20 mM *N*-ethylmaleimide (Sigma-Aldrich) at 36 h postinfection. Samples were subjected to immunoprecipitation assay as described above with anti-NS3 or anti-NS5 monoclonal antibody. Western blotting was subsequently performed with anti-NS3, NS5, and K48-polyubiquitin antibodies.

### *In vitro* transcription of JEV replicon and replication analysis.

The plasmid carrying the JEV subgenomic replicon fused with a luciferase reporter gene was transcribed *in vitro* using the T7 MEGAscript kit (Ambion) according to the manufacturer's instructions. The transcribed JEV replicon RNA was transfected into HEK293T cells using the Lipofectamine 2000 (Invitrogen). At 36 h posttransfection, the cells were harvested, and the replication of JEV replicon was analyzed by quantitative real-time PCR and Western blotting.

### Statistical analysis.

All results are presented as the mean ± SEM. Statistical significance was determined by using a Student *t* test, and a *P* value <0.05 was considered statistically significant.

## RESULTS

### JEV infection downregulates miR-33a-5p expression.

**FIG 1:**
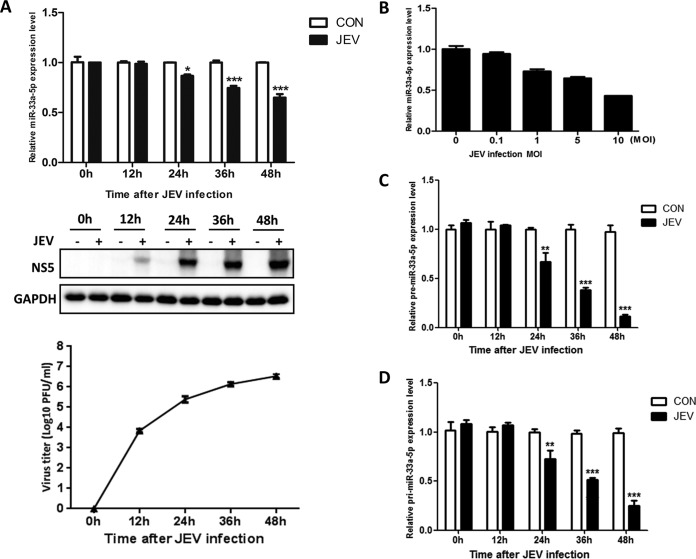
JEV infection downregulates miR-33a-5p expression. (A) HEK293T cells were infected with JEV at an MOI of 1.0. Cells were harvested at 0, 12, 24, 36, and 48 h postinfection, respectively. Total cellular RNA was extracted and subjected to RT reaction. The level of miR-33a-5p was determined by quantitative real-time PCR (upper panel). Western blotting was performed to examine the expression of JEV NS5 protein (lower panel). GAPDH (glyceraldehyde-3-phosphate dehydrogenase) expression was verified as loading control. The corresponding virus titer was measured by plaque assay. (B) HEK293T cells were infected with JEV at the indicated MOIs. Cells were harvested at 36 h postinfection, and quantitative real-time PCR was performed to determine the expression of miR-33a-5p. (C and D) HEK293T cells were infected with JEV at an MOI of 1.0. Cells were harvested at 0, 12, 24, 36, and 48 h postinfection, respectively. Total cellular RNA was extracted and subjected to RT reaction. The level of pre-miR-33a-5p (C) and pri-miR-33a-5p (D) were determined by quantitative real-time PCR. Data are shown as means ± the standard errors of the mean (SEM) of at least three independent experiments, with the error bars representing the standard deviations. *, *P* < 0.05; ***, *P* < 0.001.

To investigate differentially expressed miRNAs during JEV infection, miRNA sequencing was performed and a large variety of up- or downregulated miRNAs was identified (see Table S1 in the supplemental material). miR-33a-5p, which showed an obviously downregulated pattern upon JEV infection and has rarely been reported to be involved in virus infections, was selected for further study. To assess the level of miR-33a-5p in HEK293T cells, the cells were infected with JEV strain P3 (MOI = 1.0) and harvested at various time points. The miR-33a-5p level was determined by quantitative real-time PCR, and the virus titer was measured by plaque assay. The miR-33a-5p level decreased over time accompanied by the increasing amount of virus titer after JEV infection (Fig. 1A). At 24 h, the miR-33a-5p level was significantly lower than in mock-infected cells (Fig. 1A). Further, we infected HEK293T cells with JEV P3 at different MOIs and found that miR-33a-5p level decreased significantly in a virus load-dependent manner (Fig. 1B). To determine miR-33a-5p expression was regulated at which step, primary (pri)- and precursor (pre)-miR-33a-5plevels were checked, and similar downward trends were observed in both of them (Fig. 1C and D). These data indicate that JEV infection downregulates miR-33a-5p expression at transcriptional level in HEK293T cells.

### miR-33a-5p inhibits JEV replication.

**FIG 2:**
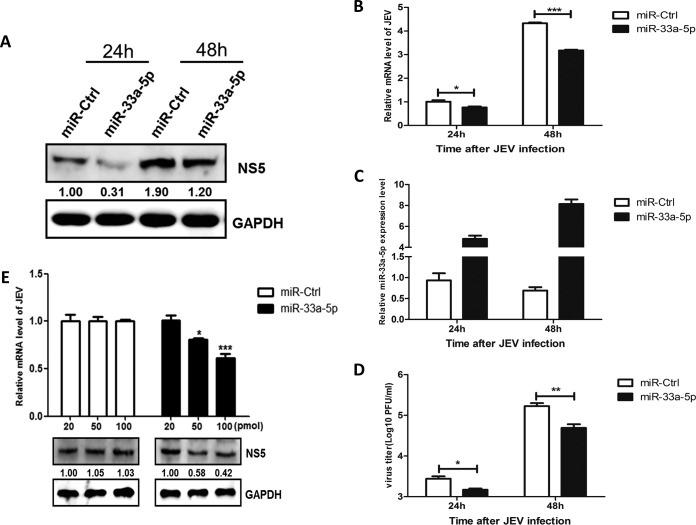
miR-33a-5p downregulates the replication of JEV. (A to D) HEK293T cells were transfected with miR-33a-5p mimics for 24 h and then infected with JEV at an MOI of 1.0. The cells were harvested at 24 and 48 h postinfection, respectively. (A) According to the previous method, JEV NS5 protein levels were determined by Western blotting and normalized to GAPDH. (B) JEV mRNA levels were determined by quantitative real-time PCR and normalized to β-actin. (C) miR-33a-5p levels were determined by quantitative real-time PCR and normalized to U6. (D) JEV titers were determined by plaque assay in BHK cells. (E) HEK293T cells were transfected with different concentrations of miR-33a-5p mimics for 24 h and then infected with JEV at an MOI of 1.0. At 48 h postinfection, the cells were collected, and JEV NS5 protein levels were determined by Western blotting and normalized to GAPDH. JEV mRNA levels were determined by quantitative real-time PCR and normalized to β-actin. Data are shown as means ± the SEM of at least three independent experiments. *, *P* < 0.05; ***, *P* < 0.001.

To test whether miR-33a-5p has a biological function in viral replication, HEK293T cells were transfected with miR-33a-5p mimic or control miRNA mimic, followed by JEV infection. Cells were harvested at different time points, and the intracellular viral NS5 level, the viral RNA and miR-33a-5p levels, and the viral titers were determined by Western blotting, quantitative real-time PCR, and plaque assays, respectively. As shown in Fig. 2, the overexpression of miR-33a-5p significantly inhibited JEV replication in a dose-dependent manner (Fig. 2E), indicating that miR-33a-5p acts as a negative regulator for JEV replication in HEK293T cells.

### EEF1A1 is a target of miR-33a-5p.

**FIG 3:**
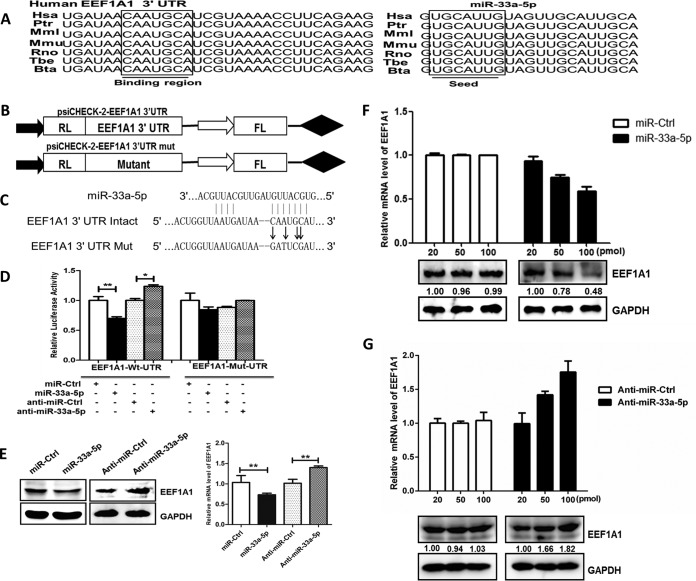
*EEF1A1* is a target of miR-33a-5p. (A) Sequence alignments of miR-33a-5p in different species and its target sites in 3′ UTR of *EEF1A1*. (B) Model of wild and mutant constructs of *EEF1A1* 3′ UTR. (C) Schematic representation of mutant reporters of *EEF1A1* 3′ UTR. The frame and bold letters indicate the point mutation. (D, E, and F) *EEF1A1* 3′ UTR is a target for miR-33a-5p. (D) Wild or mutant reporter constructs of *EEF1A1* 3′ UTR were cotransfected with indicated oligonucleotides into HEK293T cells. After 24 h, the Renilla and firefly luciferase activities were assayed. (E) HEK293T cells were transfected with miR-33a-5p mimics or miR-33a-5p inhibitor for 48 h, and the *EEF1A1* mRNA and protein expression levels were determined by quantitative real-time PCR (normalized to β-actin) and Western blotting (normalized to GAPDH), respectively. (F and G) HEK293T cells were transfected with different concentrations of miR-33a-5p mimics (F) or miR-33a-5p inhibitors (G) for 24 h and then infected with JEV at an MOI of 1.0. At 48 h postinfection, the EEF1A1 protein levels were determined by Western blotting and normalized to GAPDH. *EEF1A1* mRNA levels were determined by quantitative real-time PCR and normalized to β-actin. Data are shown as means ± SEM of at least three independent experiments. *, *P* < 0.05; ***, *P* < 0.001.

We used publically available miRNA target-prediction algorithms TargetScan, PIT, Pictar, and miRanda to identify miR-33a-5p targets with potential relevance to the regulation of JEV replication. The potential target *EEF1A1* was singled out for further study because its encoded protein is an important eukaryotic factor that participates in the replication of a variety of viruses (22–25). The sequences of miR-33a-5p and its target site in the 3′ UTR of *EEF1A1* were aligned with those from different species, and these sequences are shown to be highly conserved among species (Fig. 3A). To determine whether *EEF1A1* mRNA is indeed a target of miR-33a-5p, we then constructed dual-luciferase reporter plasmids carrying the *EEF1A1* 3′ UTR with the wild-type or base pair mutant miR-33a-5p binding region (Fig. 3B and C). The luciferase activity markedly decreased when cells were cotransfected with the miR-33a-5p mimic and wild-type *EEF1A1* 3′-UTR luciferase reporter. In contrast, the luciferase activity increased after treatment with miR-33a-5p inhibitors. To confirm that this reduction in luciferase activity was indeed due to interaction of miR-33a-5p with the 3′ UTR of *EEF1A1*, a mutant dual luciferase reporter containing four base pair mutations in the seed region was also cotransfected into HEK293T cells, together with miR-33a-5p mimics or inhibitors (Fig. 3D). As expected, no significant effect of either miR-33a-5p mimics or inhibitors was observed (Fig. 3D). To further validate the impact of interaction between miR-33a-5p and the *EEF1A1* 3′ UTR, expression of endogenous *EEF1A1* was measured in HEK293T cells treated with miR-33a-5p mimics or inhibitors. Overexpression of miR-33a-5p resulted in a significant reduction in *EEF1A1* at both transcriptional and posttranscriptional levels, whereas the application of miR-33a-5p inhibitor restored the expression of *EEF1A1* (Fig. 3E to G). These results suggested that *EEF1A1* is a direct target of miR-33a-5p.

### JEV infection upregulates *EEF1A1* expression.

**FIG 4:**
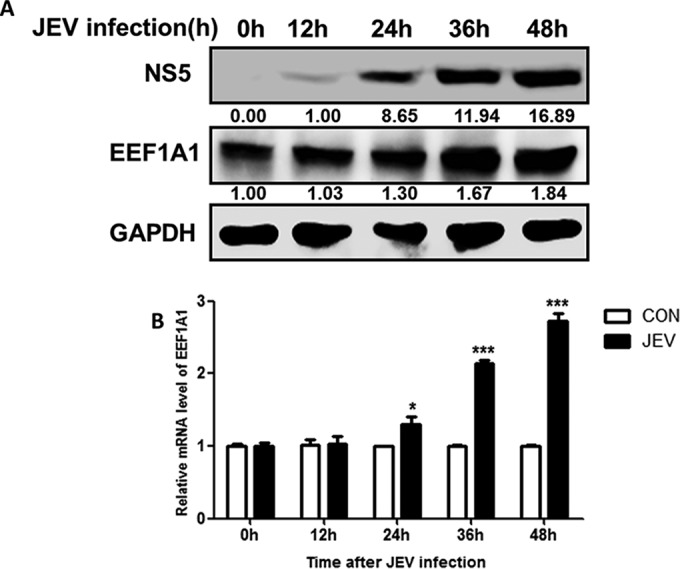
JEV infection upregulates *EEF1A1* expression. (A and B) HEK293T cells were mock infected or infected with JEV at an MOI of 1.0. At different times postinfection, as indicated, *EEF1A1* protein levels were determined by Western blotting and normalized to GAPDH. The intracellular *EEF1A1* mRNA levels (the relative *EEF1A1* mRNA levels were quantified compared to the levels of the control groups. The values from the control groups were determined by quantitative real-time PCR and normalized to β-actin. Data are shown as means ± the SEM of at least three independent experiments. *, *P* < 0.05; ***, *P* < 0.001.

Having shown that JEV infection downregulates miR-33a-5p expression and that miR-33a-5p targets *EEF1A1* mRNA, we hypothesized that JEV infection may upregulate *EEF1A1* expression. To address this possibility, HEK293T cells were infected with JEV P3 (MOI = 1.0), and cells were harvested at different time points. Quantitative real-time PCR and Western blotting showed that *EEF1A1* mRNA and protein levels were significantly upregulated in JEV-infected cells in a time-dependent manner (Fig. 4).

### EEF1A1 positively regulates the replication of JEV.

**FIG 5:**
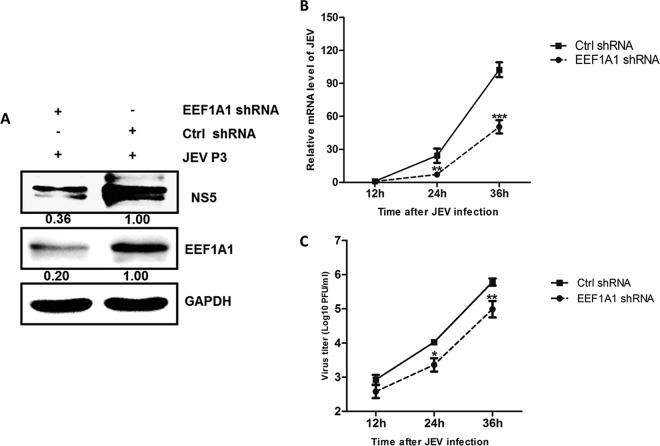
*EEF1A1* positively regulates the replication of JEV. (A) HEK293T cells were transfected with *EEF1A1*-shRNA or control shRNA for 24 h, and then cells were infected with JEV at an MOI of 1.0. Cells were collected at 48 h postinfection, and *EEF1A1* and JEV NS5 protein levels were determined by Western blotting and normalized to GAPDH. (B and C) HEK293T cells were transfected with *EEF1A1*-shRNA or control shRNA for 24 h, and cells were subsequently infected with JEV at an MOI of 1.0. Cells were harvested at 12, 24, and 36 h postinfection, respectively. (B) *EEF1A1* and JEV mRNA levels were determined by quantitative real-time PCR and normalized to β-actin. (C) JEV titers were determined by plaque assay in BHK cells. Data are shown as means ± the SEM of at least three independent experiments. *, *P* < 0.05; ***, *P* < 0.001.

To assess whether *EEF1A1* is critical for JEV replication, we silenced *EEF1A1* with *EEF1A1*-specific shRNA and verified the effects on JEV replication. HEK293T cells transfected with sh-*EEF1A1* or sh-NC were infected with JEV. The viral protein and mRNA levels and viral titers were determined by Western blotting, quantitative real-time PCR, and plaque assays, respectively. As shown in Fig. 5A, *EEF1A1* shRNA significantly inhibited the endogenous expression of *EEF1A1*. In *EEF1A1*-knockdown cells, viral protein levels were dramatically decreased (Fig. 5A), and viral mRNA levels were reduced by ∼22, 40, and 50% at 12, 24, and 36 h postinfection, respectively (Fig. 5B). The viral titers were also found to be decreased markedly (Fig. 5C). Taken together, these results demonstrated a positive regulatory role for *EEF1A1* in JEV replication.

### EEF1A1 interacts with components of the JEV replicase complex.

**FIG 6:**
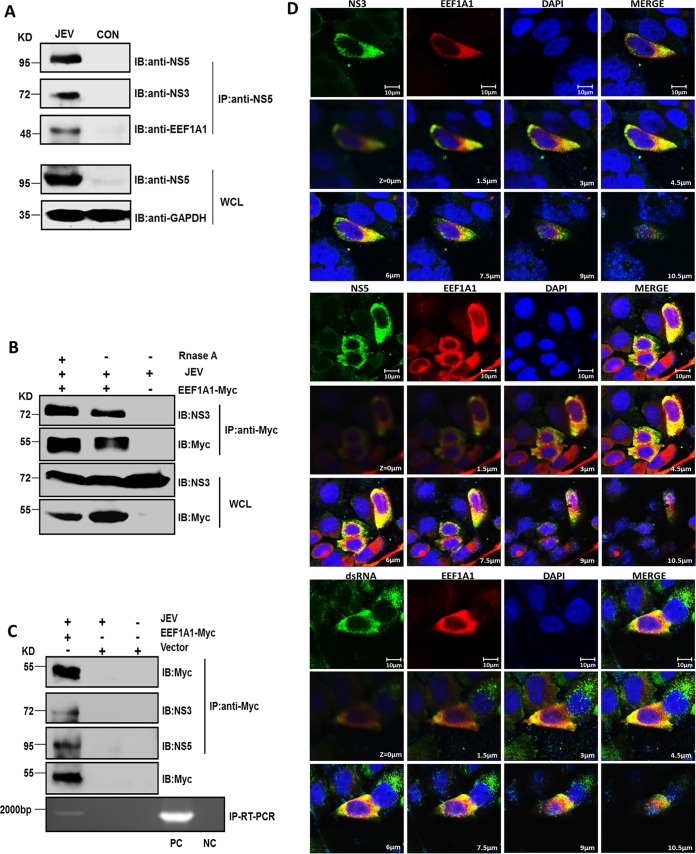
*EEF1A1* interacts with the components of the JEV replicase complex. (A) Coimmunoprecipitation identification of interaction between endogenous *EEF1A1* and JEV NS3, NS5. HEK293T cells were infected with JEV at an MOI of 1.0, and cell lysates were harvested at 36 h postinfection for coimmunoprecipitation with anti-NS5 antibodies. The precipitates were then analyzed by Western blotting with anti-NS5, anti-NS3, or anti-*EEF1A1* antibodies. (B) HEK293T cells were transfected with Myc-tagged *EEF1A1*plasmid or vector; 24 h later, the cells were infected with JEV at an MOI of 1.0. Cell lysates treated with or without RNase A were harvested at 36 h postinfection for coimmunoprecipitation with anti-Myc antibodies. The precipitates were then analyzed by Western blotting with anti-NS3 or anti-Myc antibodies. (C) Interaction of *EEF1A1* with NS3, NS5, and viral RNA in JEV-infected cells. HEK293T cells were transfected with Myc-tagged *EEF1A1* plasmid or vector; 24 h later, the cells were infected with JEV at an MOI of 1.0. Cell lysates were harvested at 36 h postinfection for coimmunoprecipitation with anti-Myc antibodies. The precipitates were then divided into two parts; one part was analyzed by Western blotting with anti-Myc, anti-NS3, and anti-NS5 antibodies, and the other was treated with TRIzol to isolate RNA and then subjected to RT-PCR to detect viral RNA. RNA extracted from cells infected or not infected with JEV was used as a positive control (PC) and as a negative control (NC), respectively. (D) Colocalization of *EEF1A1* with JEV NS3, NS5, and viral dsRNA. HEK293T cells were transfected with Myc-tagged *EEF1A1* plasmid and, 24 h later, infected with JEV at an MOI of 1.0. At 36 h postinfection, the cells were fixed with ice-cold methanol and subjected to indirect immunofluorescence analysis. JEV NS3, NS5, and viral dsRNA were stained green, *EEF1A1* was stained red, and nuclei were stained blue. The cells were observed using a confocal microscope (Zeiss). Scale bar, 10 μm.

*EEF1A1* is important for West Nile virus and dengue virus minus-strand RNA synthesis through interactions with viral RNA and replication complex proteins, including NS3 and NS5 (26). Because West Nile virus belongs to the JEV serocomplex, we hypothesized that *EEF1A1* may play a similar role in JEV replication. We have also previously reported an interaction between *EEF1A1* and JEV NS5 that enhances the stability of JEV replicase complex (20). Therefore, we investigated whether *EEF1A1* also interacts with JEV NS3. First, HEK293T cells were infected with JEV, and cell lysates were collected at 36 h postinfection. Anti-NS5 antibody was used to perform an immunoprecipitation assay. We found that, in addition to NS5, anti-NS5 antibody can also pull down NS3 and *EEF1A1*, which indicated that *EEF1A1* also interacts with JEV NS3, and this interaction has no relationship with *EEF1A1* overexpression (Fig. 6A). Furthermore, we demonstrated that the interaction between EEF1A1and NS3 was not mediated by RNA, since RNase A treatment prior to the coimmunoprecipitation assays did not diminish the interaction (Fig. 6B). The viral replication complex contains not only proteins involved in genome replication, but also viral double-stranded RNA (dsRNA), an RNA intermediate in replication of several positive-strand RNA viruses, including JEV. In order to validate the association of *EEFIA1* with the JEV replicase complex, the immunoprecipitation assay and IP-RT-PCR were performed with JEV- or mock-infected HEK293T cells. The results showed the coprecipitation of *EEF1A1* with NS3, NS5, and viral RNA in JEV-infected cells (Fig. 6C). We further examined the intracellular colocalization of *EEF1A1* with NS3, NS5, and viral dsRNA in JEV-infected HEK293T cells by immunofluorescence analysis. Through multiple layers image scanning, a strong colocalization of *EEF1A1* with NS5, NS3, and dsRNA was observed in JEV-infected cells, which corroborated the presence of *EEF1A1* in the JEV replicase complex (Fig. 6D). These results suggest that *EEF1A1* can convincingly interact with JEV replication components.

### EEF1A1 stabilizes the components of viral replicase complex.

**FIG 7:**
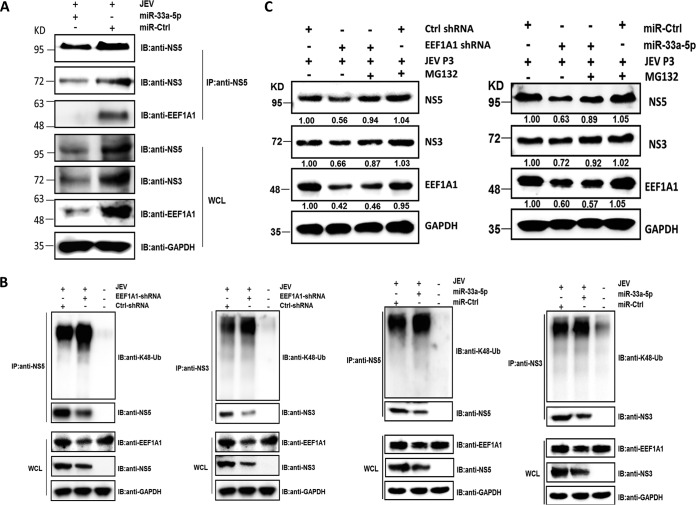
*EEF1A1* stabilizes components of the viral replicase complex. (A) *EEF1A1* enhances JEV replication by increasing the interaction amount between it and viral replicase components. HEK293T cells were transfected with miR-33a-5p mimics or control mimics and, 24 h later, the cells were infected with JEV at an MOI of 1.0. Cell lysates were harvested at 36 h postinfection, and protein concentrations were measured and quantified. The cell lysates were then subjected to coimmunoprecipitation with anti-NS5 antibodies. The precipitates were analyzed by Western blotting with anti-NS3, anti-NS5, or anti-*EEF1A1* antibodies. (B) HEK293T cells containing control shRNA or *EEF1A1* shRNA, or HEK293T cells containing miR-33a-5p mimics or control mimics, were infected with JEV at an MOI of 1.0. Cell lysates were harvested at 36 h posttransfection and immunoprecipitated with anti-NS3 or anti-NS5 antibodies. The precipitates were subjected to Western blotting with anti-NS3, anti-NS5, and anti-K48-ubiquitin antibodies. (C) HEK293T cells containing control shRNA or *EEF1A1* shRNA, or HEK293T cells containing miR-33a-5p mimics or control mimics were infected with JEV at an MOI of 1.0. In one set of experiments, 20 nM MG132 was added at 24 h postinfection to inhibit proteasomal degradation. Cell lysates were prepared at 12 h after treatment and analyzed by Western blotting with anti-NS3, anti-NS5, and anti-*EEF1A1* antibodies.

*EEF1A1* exhibits a chaperone-like activity, and it has not only been shown to interact with an unfolded protein after its release from the ribosome but also been investigated to mediate protein refolding (27–30). This prompted us that *EEF1A1* may augment the stability of viral proteins through its chaperone-like activity. To validate this hypothesis, anti-NS5 antibody was used to pull down the NS5-containing replicase complex from JEV-infected cells transfected with miR-33a-5p mimics. It was found that overexpression of miR-33a-5p significantly decreased the interaction amount between *EEF1A1* and viral replicase components (Fig. 7A). Furthermore, we subsequently checked the effect of *EEF1A1* on the Lys48 (K48) ubiquitination of NS3 and NS5, which serves as the essential sign of protein degradation mediated by the ubiquitin-proteasome system. NS3 and NS5 proteins were immunoprecipitated from the JEV-infected cells that were initially transfected with *EEF1A1* shRNA, miR-33a-5p mimics or their controls and then subjected to Western blotting with anti-NS3, anti-NS5, and anti-K48-ubiquitin antibodies. It was found that knockdown of *EEF1A1* significantly increased the K48 ubiquitination of NS3 and NS5 (Fig. 7B), suggesting that downregulation of *EEF1A1* leads to proteasomal degradation of NS3 and NS5 proteins.

To further validate this mechanism, JEV-infected *EEF1A1*-knockdown cells or miR-33a-5p-overexpressed cells were treated with proteasomal inhibitor, MG132. It was observed that the protein products of JEV NS3 and NS5 were obviously recovered by MG132 treatment (Fig. 7C). Taken together, these data suggest that *EEF1A1* interacts with the viral proteins in the replicase complex and protects them from being degraded through the ubiquitin-proteasome system and thus benefits the viral replication.

### miR-33a-5p-mediated regulation of JEV replication is achieved through targeting *EEF1A1*.

**FIG 8:**
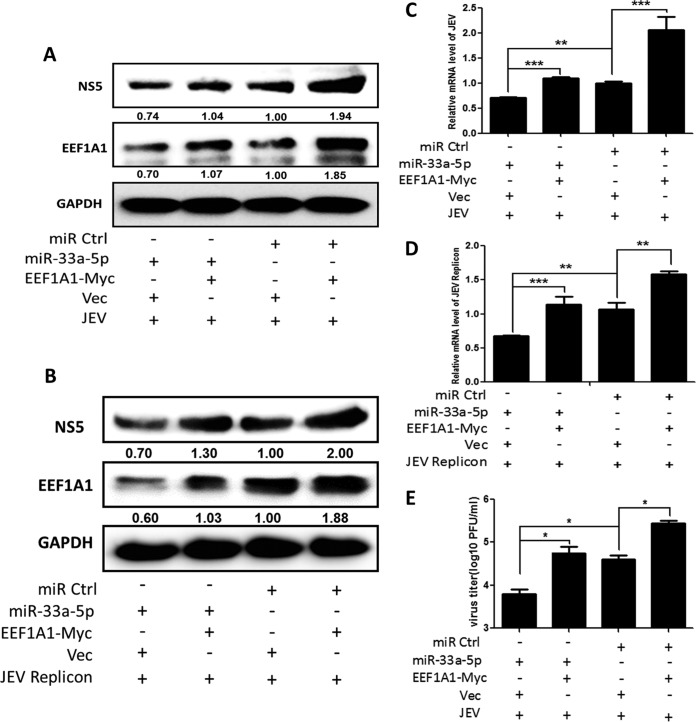
miR-33a-5p-mediated regulation of JEV replication is achieved through targeting *EEF1A1*. (A to E) HEK293T cells were cotransfected with miR-33a-5p mimics or control miRNA mimics and Myc-tagged *EEF1A1* plasmid or vector for 24 h, and infected with JEV at an MOI of 1.0 or transfected with JEV replicon, then cells were harvested at 36 h postinfection or transfection. (A and B) According to the previous method, JEV NS5 and *EEF1A1* protein levels were determined by Western blotting and normalized to GAPDH. (C and D) JEV mRNA levels were determined by quantitative real-time PCR and normalized to β-actin. (E) JEV titers were determined by plaque assay in BHK cells. Data are shown as means ± the SEM of at least three independent experiments. *, *P* < 0.05; ***, *P* < 0.001.

To fully illustrate the relationship among miR-33a-5p, *EEF1A1*, and JEV, HEK293T cells were cotransfected with miR-33a-5p mimics or control miRNA mimics and Myc-tagged *EEF1A1* plasmid or vector, followed by JEV infection or JEV replicon transfection. Cells were harvested at 36 h postinfection or transfection, and the intracellular viral NS5 level, the RNA levels, and the viral titers were determined by Western blotting, quantitative real-time PCR, and plaque assays, respectively. As shown in Fig. 8, cotransfection of miR-33a-5p mimics and vector markedly reduced JEV replication, whereas cotransfection of miR-33a-5p mimics and Myc-tagged *EEF1A1* plasmid countervailed the inhibition, indicating that miR-33a-5p negatively regulates JEV replication by targeting *EEF1A1*.

## DISCUSSION

As regulators of gene expression at the posttranscriptional level, miRNAs play critical roles in many cellular processes. Viral infection alters the miRNA expression profile in the host cells, and the altered miRNAs can, in turn, affect the virus life cycle (31, 32). Increasing evidence suggests a vital role for miRNAs during virus infection (2, 33–36). However, for JEV—an important zoonotic flavivirus—very little is known about how miRNAs participate in infection. Pareek et al. reported that induction of miR-155 in human microglial cells may negatively modulate JEV-induced innate immune gene expression and may have a beneficial role in limiting JEV replication (37). Thounaojam et al. found that miR-29b can modulate JEV-induced microglia activation by targeting tumor necrosis factor α-induced protein 3 (38). Sharma et al. found that upregulation of miR-146a by JEV led to suppression of nuclear factor-κB activity and disruption of antiviral Janus kinase-signal transducer and activator of transcription signaling, which helps the virus to evade the host immune response (39). In addition, we also reported that upregulation of miR-15b by JEV can modulate JEV-mediated inflammatory response via targeting ring finger protein 125 (8).

Through miRNA sequencing, we identified miR-33a-5p as our research focus. miR-33a-5p is one of the mature forms of miR-33a. Many studies have reported functions for miR-33a, but most focus on its involvement in lipid and glucose metabolism, cancer development, and the regulation of cell proliferation (40–43). Relatively little has been reported about the involvement of miR-33a in viral infections. Although miR-33a participates in HBV and HCV infection, this has been attributed to its involvement in fatty acid metabolism (44, 45). Recent studies have demonstrated that human miR-33a-5p overexpression significantly reduces HIV particle production in MT2 and primary T CD4^+^ cells, indicating that miR-33a-5p plays an important role in HIV infection (46). In our study, we found that human miR-33a-5p also plays a critical role in JEV infection.

MiR-33a-5p expression was downregulated after JEV infection, but the difference was not significant compared to the control in the first 12 h, indicating that miR-33a-5p exerts its function mainly at the late stages of JEV infection. miR-33a is an intronic microRNA that coordinately expressed with its host gene SREBP-2. In each case, changes in miR-33a expression were closely paralleled by changes in SREBP-2 mRNA (41, 43). In our study, we observed that SREBP-2 mRNA level was also downregulated after JEV infection (data not shown), showing the same trend as miR-33a-5p. In addition to confirming that miR-33a-5p is downregulated after JEV infection, this result suggests a potential mechanism by which JEV downregulates miR-33a-5p expression.

In previous studies, miR-33a-5p was mainly shown to target the cellular cholesterol efflux transporter ATP-binding cassette transporter A1, which plays an important role in lipid metabolism (43, 47–49). Here, we identified *EEF1A1* as a novel target of miR-33a-5p that is upregulated by JEV infection in a time-dependent manner. *EEF1A1* is an important, well-characterized eukaryotic protein, which has a well-defined role in protein synthesis. It binds to GTP and transfers the aminoacylated-tRNAs to the A site of ribosome during translation elongation. In addition, Recent studies also identified the crucial role of EEF1A1 in infections of several viruses such as West Nile virus, dengue virus, and turnip yellow mosaic virus (22–25). In this study, we demonstrated that *EEF1A1* can interact with components of the JEV replicase complex and facilitates JEV replication. Given that EEF1A1 also exhibits a chaperone-like activity, we investigated the possible role of EEF1A1 in stabilizing JEV replication complex. We found that EEF1A1 can protect NS3 and NS5 from being degraded through ubiquitin-proteasome system. The data highlight only one possible mechanism by which EEF1A1 regulates JEV replication; however, the other mechanisms are still needed to be explored. In this study, we also examined the role of EEF1A1 in the replication of JEV replicon, which excludes the effect of EEF1A1 on virus entry and assembly processes, but whether EEF1A1 affects viral replication through regulating RNA replication or stimulating virus-specific translation remains unclear. To our knowledge, this is the first study to demonstrate the importance of *EEF1A1* in JEV replication and the regulation of *EEF1A1* via miR-33a-5p. Except for EEF1A1, JEV has also been reported to regulate the expression of multiple host proteins, such as heat shock protein 70 (HSP70) (20), interferon-induced transmembrane protein 3 (IFITM3), and Ran-binding protein 2 (RANBP2) (50). All of these proteins can modify JEV replication, which indicates a common strategy for virus to facilitate its own replication.

**FIG 9:**
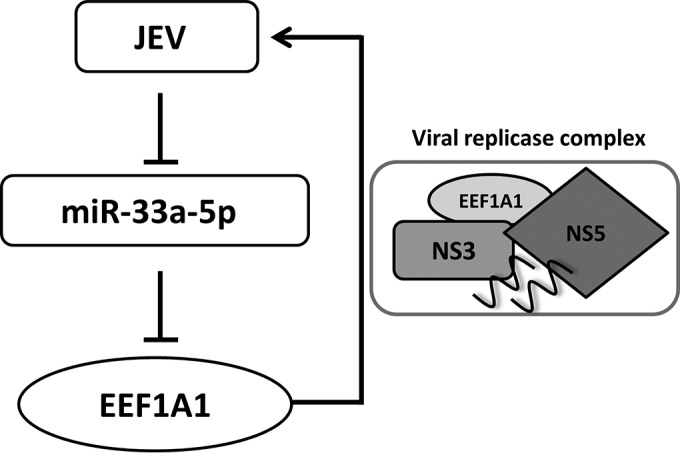
Proposed model of regulatory role of miR-33a-5p in JEV infection. JEV infection inhibits miR-33a-5p expression and maturation, whereas miR-33a-5p targets *EEF1A1*, so in JEV-infected cells *EEF1A1* was upregulated. *EEF1A1* can interact with viral replicase components and, through the interaction, *EEF1A1* stabilizes the components of viral replicase complex, which ultimately facilitates viral replication.

In summary, this study showed that miR-33a-5p level was downregulated after JEV infection, and EEF1A1 was identified as a novel target of miR-33a-5p. Our results also demonstrated that EEF1A1 can interact with components of the JEV replicase complex and positively regulate JEV replication (Fig. 9). These findings provide us a better understanding of JEV replication mechanism.

## Supplementary Material

Supplemental material
