# Supplementary material for: MicroRNA-33a-5p Modulates Japanese Encephalitis Virus Replication by Targeting Eukaryotic Translation Elongation Factor 1A1
Source: J Virol. 2016 Mar 11;90(7):3722–34. doi: 10.1128/JVI.03242-15 (PMC4794666; doi:10.1128/JVI.03242-15)
Supplement: Supplemental material [file supp_90_7_3722__index.html]

Supplemental material 

# MicroRNA-33a-5p Modulates Japanese Encephalitis Virus Replication by Targeting Eukaryotic Translation Elongation Factor 1A1

## Supplemental material

- Supplemental file 1 -

  Table S1 (MiRNA profile results.)

  PDF, 749K
