# Supplementary material for: MicroRNA-33a-5p Modulates Japanese Encephalitis Virus Replication by Targeting Eukaryotic Translation Elongation Factor 1A1
Source: J Virol. 2016 Mar 11;90(7):3722–34. doi: 10.1128/JVI.03242-15 (PMC4794666; doi:10.1128/JVI.03242-15)
Supplement: Supplemental material [file JVI.03242-15_zjv999091514so1.pdf]

con （control group）;P3(JEV P3 group)

pairwise	miR-name	RNA-con-total-reads	RNA-P3-total-reads	RNA-con-expressed	RNA-P3-expressed	RNA-con-std	RNA-P3-std	fold-change(log2 RNA-P3/RNA-con)	p-value	sig-lable
RNA-con-RNA-P3	hsa-miR-191-3p	15617308	17985385	26	2	1.6648	0.1112	-3.90412018	2.56E-07	**
RNA-con-RNA-P3	hsa-miR-4448	15617308	17985385	21	2	1.3447	0.1112	-3.59605565	8.14E-06	**
RNA-con-RNA-P3	hsa-miR-145-3p	15617308	17985385	16	2	1.0245	0.1112	-3.20369129	0.0002376	**
RNA-con-RNA-P3	hsa-miR-143-5p	15617308	17985385	107	14	6.8514	0.7784	-3.1378152	3.50E-22	**
RNA-con-RNA-P3	hsa-miR-3138	15617308	17985385	30	4	1.9209	0.2224	-3.11055372	4.64E-07	**
RNA-con-RNA-P3	hsa-miR-1299	15617308	17985385	37	5	2.3692	0.278	-3.0912432	2.15E-08	**
RNA-con-RNA-P3	hsa-miR-149-3p	15617308	17985385	37	5	2.3692	0.278	-3.0912432	2.15E-08	**
RNA-con-RNA-P3	hsa-miR-551b-5p	15617308	17985385	71	11	4.5462	0.6116	-2.89400084	4.20E-14	**
RNA-con-RNA-P3	hsa-miR-204-3p	15617308	17985385	542	91	34.705	5.0597	-2.77802393	7.65E-94	**
RNA-con-RNA-P3	hsa-miR-642a-3p	15617308	17985385	55	10	3.5217	0.556	-2.66311523	2.22E-10	**
RNA-con-RNA-P3	hsa-miR-23b-5p	15617308	17985385	4083	747	261.44	41.5337	-2.65412945	0	**
RNA-con-RNA-P3	hsa-miR-1228-5p	15617308	17985385	19	4	1.2166	0.2224	-2.45162622	0.0004576	**
RNA-con-RNA-P3	hsa-miR-450a-3p	15617308	17985385	50	11	3.2016	0.6116	-2.38813276	1.37E-08	**
RNA-con-RNA-P3	hsa-miR-758-5p	15617308	17985385	203	46	12.998	2.5576	-2.3454715	4.67E-30	**
RNA-con-RNA-P3	hsa-miR-1268a	15617308	17985385	4468	1053	286.09	58.5475	-2.28880372	0	**
RNA-con-RNA-P3	hsa-miR-1323	15617308	17985385	144	34	9.2205	1.8904	-2.28615345	3.88E-21	**
RNA-con-RNA-P3	hsa-miR-944	15617308	17985385	59	14	3.7779	0.7784	-2.2790009	1.94E-09	**
RNA-con-RNA-P3	hsa-miR-1268b	15617308	17985385	4709	1152	301.52	64.052	-2.23495923	0	**
RNA-con-RNA-P3	hsa-miR-769-5p	15617308	17985385	20	5	1.2806	0.278	-2.20366313	0.0007309	**
RNA-con-RNA-P3	hsa-miR-33a-5p*	15617308	17985385	2358	604	150.99	33.5828	-2.16862322	2.76E-300	**
RNA-con-RNA-P3	hsa-miR-181c-3p	15617308	17985385	37	10	2.3692	0.556	-2.0912432	7.28E-06	**
RNA-con-RNA-P3	hsa-miR-181b-3p	15617308	17985385	70	19	4.4822	1.0564	-2.08505082	6.09E-10	**
RNA-con-RNA-P3	hsa-miR-33b-3p	15617308	17985385	25	7	1.6008	0.3892	-2.04020946	0.0003053	**
RNA-con-RNA-P3	hsa-let-7e-5p	15617308	17985385	4E+05	1E+05	27260	6713.84	-2.02159642	0	**
RNA-con-RNA-P3	hsa-miR-3928	15617308	17985385	152	44	9.7328	2.4464	-1.9921946	6.91E-19	**
RNA-con-RNA-P3	hsa-miR-301a-5p	15617308	17985385	230	71	14.727	3.9476	-1.89944524	3.57E-26	**
RNA-con-RNA-P3	hsa-miR-3130-3p	15617308	17985385	45	14	2.8814	0.7784	-1.88818634	3.62E-06	**
RNA-con-RNA-P3	hsa-miR-1908	15617308	17985385	16	5	1.0245	0.278	-1.8817632	0.0065815	**
RNA-con-RNA-P3	hsa-miR-3141	15617308	17985385	151	48	9.6688	2.6688	-1.85714565	3.25E-17	**
RNA-con-RNA-P3	hsa-miR-185-3p	15617308	17985385	142	47	9.0925	2.6132	-1.79885948	1.22E-15	**
RNA-con-RNA-P3	hsa-miR-504	15617308	17985385	54	18	3.4577	1.0008	-1.78865901	9.79E-07	**
RNA-con-RNA-P3	hsa-miR-744-5p	15617308	17985385	5983	2032	383.1	112.981	-1.76164822	0	**
RNA-con-RNA-P3	hsa-miR-124-3p	15617308	17985385	50	17	3.2016	0.9452	-1.76010154	3.22E-06	**

RNA-con-RNA-P3	hsa-miR-184	15617308	17985385	20	7	1.2806	0.3892	-1.7182363	0.0041404	**
RNA-con-RNA-P3	hsa-miR-3190-3p	15617308	17985385	20	7	1.2806	0.3892	-1.7182363	0.0041404	**
RNA-con-RNA-P3	hsa-miR-548ai	15617308	17985385	20	7	1.2806	0.3892	-1.7182363	0.0041404	**
RNA-con-RNA-P3	hsa-miR-570-5p	15617308	17985385	20	7	1.2806	0.3892	-1.7182363	0.0041404	**
RNA-con-RNA-P3	hsa-miR-4450	15617308	17985385	17	6	1.0885	0.3336	-1.70615021	0.0087133	**
RNA-con-RNA-P3	hsa-miR-185-5p	15617308	17985385	31747	11244	2032.8	625.174	-1.70114408	0	**
RNA-con-RNA-P3	hsa-miR-877-5p	15617308	17985385	140	50	8.9644	2.78	-1.68912214	2.95E-14	**
RNA-con-RNA-P3	hsa-miR-654-3p	15617308	17985385	131	48	8.3881	2.6688	-1.65215287	4.65E-13	**
RNA-con-RNA-P3	hsa-miR-940	15617308	17985385	30	11	1.9209	0.6116	-1.6511221	0.0005979	**
RNA-con-RNA-P3	hsa-miR-128	15617308	17985385	12874	4777	824.34	265.605	-1.63396306	0	**
RNA-con-RNA-P3	hsa-miR-548j	15617308	17985385	35	13	2.2411	0.7228	-1.63253861	0.0002304	**
RNA-con-RNA-P3	hsa-miR-628-5p	15617308	17985385	50	19	3.2016	1.0564	-1.59963687	1.36E-05	**
RNA-con-RNA-P3	hsa-miR-1307-5p	15617308	17985385	388	148	24.844	8.2289	-1.59413759	5.55E-34	**
RNA-con-RNA-P3	hsa-miR-152	15617308	17985385	3682	1406	235.76	78.1746	-1.59257222	9.73E-307	**
RNA-con-RNA-P3	hsa-miR-99a-3p	15617308	17985385	231	89	14.791	4.9485	-1.57968567	1.28E-20	**
RNA-con-RNA-P3	hsa-miR-380-5p	15617308	17985385	18	7	1.1526	0.3892	-1.56630831	0.0110017	*
RNA-con-RNA-P3	hsa-miR-5010-5p	15617308	17985385	18	7	1.1526	0.3892	-1.56630831	0.0110017	*
RNA-con-RNA-P3	hsa-miR-217	15617308	17985385	95	37	6.083	2.0572	-1.56410095	3.31E-09	**
RNA-con-RNA-P3	hsa-miR-4435	15617308	17985385	33	13	2.113	0.7228	-1.54762436	0.0005843	**
RNA-con-RNA-P3	hsa-miR-32-5p	15617308	17985385	147	58	9.4126	3.2248	-1.5453836	2.84E-13	**
RNA-con-RNA-P3	hsa-miR-1277-5p	15617308	17985385	30	12	1.9209	0.6672	-1.52559122	0.0011888	**
RNA-con-RNA-P3	hsa-miR-3690	15617308	17985385	20	8	1.2806	0.4448	-1.52559122	0.0084434	**
RNA-con-RNA-P3	hsa-miR-4425	15617308	17985385	115	47	7.3636	2.6132	-1.49459372	3.04E-10	**
RNA-con-RNA-P3	hsa-miR-99b-3p	15617308	17985385	1416	580	90.669	32.2484	-1.49137543	2.43E-108	**
RNA-con-RNA-P3	hsa-miR-105-3p	15617308	17985385	36	15	2.3051	0.834	-1.46671005	0.0005512	**
RNA-con-RNA-P3	hsa-miR-628-3p	15617308	17985385	64	27	4.098	1.5012	-1.44880379	4.65E-06	**
RNA-con-RNA-P3	hsa-miR-320a	15617308	17985385	1E+05	63157	9298	3511.57	-1.40479559	0	**
RNA-con-RNA-P3	hsa-let-7c	15617308	17985385	6E+05	3E+05	41079	15543.4	-1.40208248	0	**
RNA-con-RNA-P3	hsa-miR-340-5p	15617308	17985385	2301	1012	147.34	56.2679	-1.38873085	3.82E-157	**
RNA-con-RNA-P3	hsa-miR-1	15617308	17985385	1756	773	112.44	42.9793	-1.38743377	3.21E-120	**
RNA-con-RNA-P3	hsa-miR-99a-5p	15617308	17985385	5940	2622	380.35	145.785	-1.3834747	0	**
RNA-con-RNA-P3	hsa-miR-660-5p	15617308	17985385	185	82	11.846	4.5593	-1.37749139	5.66E-14	**
RNA-con-RNA-P3	hsa-miR-1306-3p	15617308	17985385	153	68	9.7968	3.7808	-1.37361905	9.24E-12	**
RNA-con-RNA-P3	hsa-miR-873-5p	15617308	17985385	199	89	12.742	4.9485	-1.36456253	9.99E-15	**
RNA-con-RNA-P3	hsa-miR-199a-3p	15617308	17985385	276	124	17.673	6.8945	-1.35800463	1.04E-19	**
RNA-con-RNA-P3	hsa-miR-199b-3p	15617308	17985385	276	124	17.673	6.8945	-1.35800463	1.04E-19	**
RNA-con-RNA-P3	hsa-miR-766-5p	15617308	17985385	82	37	5.2506	2.0572	-1.35180023	8.54E-07	**
RNA-con-RNA-P3	hsa-let-7b-5p	15617308	17985385	3E+06	1E+06	168958	66450.7	-1.34630662	0	**
RNA-con-RNA-P3	hsa-miR-299-3p	15617308	17985385	75	34	4.8024	1.8904	-1.34506404	2.76E-06	**
RNA-con-RNA-P3	hsa-miR-1287	15617308	17985385	125	58	8.0039	3.2248	-1.31149345	2.78E-09	**
RNA-con-RNA-P3	hsa-miR-1285-3p	15617308	17985385	209	97	13.383	5.3933	-1.31111824	1.46E-14	**

RNA-con-RNA-P3	hsa-miR-548e	15617308	17985385	90	42	5.7628	2.3352	-1.30322384	5.31E-07	**
RNA-con-RNA-P3	hsa-miR-27b-3p	15617308	17985385	3329	1585	213.16	88.1271	-1.27428519	1.99E-198	**
RNA-con-RNA-P3	hsa-miR-876-5p	15617308	17985385	23	11	1.4727	0.6116	-1.26780326	0.0138535	*
RNA-con-RNA-P3	hsa-let-7d-5p	15617308	17985385	72220	34587	4624.4	1923.06	-1.2658478	0	**
RNA-con-RNA-P3	hsa-miR-130b-5p	15617308	17985385	25	12	1.6008	0.6672	-1.26260188	0.0104737	*
RNA-con-RNA-P3	hsa-miR-584-5p	15617308	17985385	1158	560	74.149	31.1364	-1.25181536	2.18E-68	**
RNA-con-RNA-P3	hsa-miR-499a-5p	15617308	17985385	62	30	3.97	1.668	-1.25101972	5.67E-05	**
RNA-con-RNA-P3	hsa-miR-589-5p	15617308	17985385	341	168	21.835	9.3409	-1.22498924	1.19E-20	**
RNA-con-RNA-P3	hsa-miR-16-1-3p	15617308	17985385	28	14	1.7929	0.7784	-1.20371141	0.0089932	**
RNA-con-RNA-P3	hsa-miR-6511a-5p	15617308	17985385	28	14	1.7929	0.7784	-1.20371141	0.0089932	**
RNA-con-RNA-P3	hsa-miR-6511b-5p	15617308	17985385	28	14	1.7929	0.7784	-1.20371141	0.0089932	**
RNA-con-RNA-P3	hsa-miR-186-3p	15617308	17985385	20	10	1.2806	0.556	-1.20366313	0.0277805	*
RNA-con-RNA-P3	hsa-miR-374b-3p	15617308	17985385	91	46	5.8269	2.5576	-1.1879379	2.76E-06	**
RNA-con-RNA-P3	hsa-miR-127-3p	15617308	17985385	5582	2835	357.42	157.628	-1.18111268	5.75E-294	**
RNA-con-RNA-P3	hsa-miR-27b-5p	15617308	17985385	900	460	57.628	25.5763	-1.17197239	2.12E-48	**
RNA-con-RNA-P3	hsa-miR-3192	15617308	17985385	131	68	8.3881	3.7808	-1.14965253	4.17E-08	**
RNA-con-RNA-P3	hsa-miR-433	15617308	17985385	636	333	40.724	18.515	-1.13718472	3.83E-33	**
RNA-con-RNA-P3	hsa-miR-1193	15617308	17985385	63	33	4.034	1.8348	-1.13658827	0.0001694	**
RNA-con-RNA-P3	hsa-miR-3679-5p	15617308	17985385	19	10	1.2166	0.556	-1.12969812	0.0415034	*
RNA-con-RNA-P3	hsa-let-7a-5p	15617308	17985385	4E+06	2E+06	246579	113452	-1.11996347	0	**
RNA-con-RNA-P3	hsa-miR-323a-5p	15617308	17985385	194	104	12.422	5.7825	-1.10314382	1.06E-10	**
RNA-con-RNA-P3	hsa-miR-330-3p	15617308	17985385	26	14	1.6648	0.7784	-1.09676525	0.0194944	*
RNA-con-RNA-P3	hsa-miR-505-5p	15617308	17985385	178	96	11.398	5.3377	-1.09443994	7.97E-10	**
RNA-con-RNA-P3	hsa-miR-432-5p	15617308	17985385	14808	8078	948.18	449.143	-1.07798589	0	**
RNA-con-RNA-P3	hsa-miR-101-3p	15617308	17985385	10560	5809	676.17	322.985	-1.06592726	0	**
RNA-con-RNA-P3	hsa-miR-98-5p	15617308	17985385	1968	1085	126.01	60.3268	-1.06271306	9.83E-89	**
RNA-con-RNA-P3	hsa-miR-379-5p	15617308	17985385	1297	719	83.049	39.9769	-1.05479446	2.08E-58	**
RNA-con-RNA-P3	hsa-miR-132-5p	15617308	17985385	18	10	1.1526	0.556	-1.05173514	0.0611879	
RNA-con-RNA-P3	hsa-miR-377-5p	15617308	17985385	132	74	8.4522	4.1145	-1.03860978	4.02E-07	**
RNA-con-RNA-P3	hsa-miR-105-5p	15617308	17985385	41	23	2.6253	1.2788	-1.03769164	0.0049139	**
RNA-con-RNA-P3	hsa-miR-323a-3p	15617308	17985385	2661	1493	170.39	83.0118	-1.03743455	9.53E-115	**
RNA-con-RNA-P3	hsa-miR-25-3p	15617308	17985385	40202	22908	2574.2	1273.7	-1.01509504	0	**
RNA-con-RNA-P3	hsa-miR-1277-3p	15617308	17985385	28	16	1.7929	0.8896	-1.01106633	0.0231996	*
RNA-con-RNA-P3	hsa-miR-664a-5p	15617308	17985385	279	160	17.865	8.8961	-1.00587485	6.89E-13	**
RNA-con-RNA-P3	hsa-miR-660-3p	15617308	17985385	33	19	2.113	1.0564	-1.00013656	0.0144751	*
RNA-con-RNA-P3	hsa-miR-183-3p	15617308	17985385	74	43	4.7383	2.3908	-0.9868761	0.0002798	
RNA-con-RNA-P3	hsa-miR-1254	15617308	17985385	24	14	1.5368	0.7784	-0.98134581	0.0405234	
RNA-con-RNA-P3	hsa-miR-20b-3p	15617308	17985385	108	63	6.9154	3.5028	-0.98130409	1.23E-05	
RNA-con-RNA-P3	hsa-miR-590-3p	15617308	17985385	41	24	2.6253	1.3344	-0.9762911	0.0074788	
RNA-con-RNA-P3	hsa-miR-196b-5p	15617308	17985385	68	40	4.3541	2.224	-0.96921775	0.000603	
RNA-con-RNA-P3	hsa-miR-218-5p	15617308	17985385	22	13	1.4087	0.7228	-0.96269599	0.053726	

RNA-con-RNA-P3	hsa-miR-449a	15617308	17985385	22	13	1.4087	0.7228	-0.96269599	0.053726	
RNA-con-RNA-P3	hsa-miR-3129-5p	15617308	17985385	98	58	6.2751	3.2248	-0.96042876	4.31E-05	
RNA-con-RNA-P3	hsa-miR-30d-5p	15617308	17985385	8327	4940	533.19	274.668	-0.95696486	2.01081025553286e-310	
RNA-con-RNA-P3	hsa-miR-486-3p	15617308	17985385	64	38	4.098	2.1128	-0.95576378	0.0010065	
RNA-con-RNA-P3	hsa-miR-374a-5p	15617308	17985385	585	348	37.458	19.349	-0.95303027	2.65E-23	
RNA-con-RNA-P3	hsa-miR-532-5p	15617308	17985385	609	370	38.995	20.5723	-0.92259345	5.39E-23	
RNA-con-RNA-P3	hsa-miR-889	15617308	17985385	2638	1608	168.92	89.4059	-0.91785721	4.31E-93	
RNA-con-RNA-P3	hsa-miR-873-3p	15617308	17985385	36	22	2.3051	1.2232	-0.91416903	0.0177315	
RNA-con-RNA-P3	hsa-miR-493-3p	15617308	17985385	289	177	18.505	9.8413	-0.91100212	1.73E-11	
RNA-con-RNA-P3	hsa-miR-3064-5p	15617308	17985385	16	10	1.0245	0.556	-0.8817632	0.1273189	
RNA-con-RNA-P3	hsa-miR-502-3p	15617308	17985385	134	84	8.5802	4.6705	-0.87743427	9.23E-06	
RNA-con-RNA-P3	hsa-miR-339-3p	15617308	17985385	497	312	31.824	17.3474	-0.87538213	1.45E-17	
RNA-con-RNA-P3	hsa-miR-590-5p	15617308	17985385	43	27	2.7534	1.5012	-0.87509802	0.0124321	
RNA-con-RNA-P3	hsa-miR-1185-5p	15617308	17985385	108	68	6.9154	3.7808	-0.87112117	7.64E-05	
RNA-con-RNA-P3	hsa-miR-654-5p	15617308	17985385	120	76	7.6838	4.2257	-0.86262962	3.54E-05	
RNA-con-RNA-P3	hsa-miR-26b-5p	15617308	17985385	4084	2593	261.5	144.173	-0.85903987	4.90E-128	
RNA-con-RNA-P3	hsa-miR-99b-5p	15617308	17985385	9004	5718	576.54	317.925	-0.85873464	1.23E-279	
RNA-con-RNA-P3	hsa-miR-130b-3p	15617308	17985385	1010	643	64.672	35.7513	-0.85514106	8.97E-33	
RNA-con-RNA-P3	hsa-miR-3152-3p	15617308	17985385	50	32	3.2016	1.7792	-0.84756438	0.0086738	
RNA-con-RNA-P3	hsa-let-7a-3p	15617308	17985385	64	41	4.098	2.2796	-0.84613929	0.0030042	
RNA-con-RNA-P3	hsa-miR-4485	15617308	17985385	106	68	6.7873	3.7808	-0.84414625	0.0001363	
RNA-con-RNA-P3	hsa-miR-151a-5p	15617308	17985385	786	508	50.329	28.2452	-0.83337848	7.99E-25	
RNA-con-RNA-P3	hsa-miR-323b-3p	15617308	17985385	136	88	8.7083	4.8929	-0.83170131	1.97E-05	
RNA-con-RNA-P3	hsa-miR-17-3p	15617308	17985385	747	484	47.832	26.9107	-0.82978112	1.65E-23	
RNA-con-RNA-P3	hsa-let-7f-5p	15617308	17985385	2E+06	1E+06	122472	69002.9	-0.82772277	0	
RNA-con-RNA-P3	hsa-miR-450b-5p	15617308	17985385	20	13	1.2806	0.7228	-0.8251515	0.1062651	
RNA-con-RNA-P3	hsa-miR-127-5p	15617308	17985385	156	102	9.9889	5.6713	-0.81664634	6.78E-06	
RNA-con-RNA-P3	hsa-miR-539-3p	15617308	17985385	217	142	13.895	7.8953	-0.81547908	1.14E-07	
RNA-con-RNA-P3	hsa-miR-2355-5p	15617308	17985385	41	27	2.6253	1.5012	-0.8063661	0.0228402	
RNA-con-RNA-P3	hsa-miR-320b	15617308	17985385	1495	987	95.727	54.8779	-0.80270213	5.95E-43	
RNA-con-RNA-P3	hsa-miR-130a-3p	15617308	17985385	2365	1564	151.43	86.9595	-0.80027837	1.44E-66	
RNA-con-RNA-P3	hsa-miR-409-3p	15617308	17985385	1167	773	74.725	42.9793	-0.79794514	1.42E-33	
RNA-con-RNA-P3	hsa-miR-548x-3p	15617308	17985385	209	139	13.383	7.7285	-0.79209809	3.87E-07	
RNA-con-RNA-P3	hsa-miR-548n	15617308	17985385	33	22	2.113	1.2232	-0.78863246	0.0453106	
RNA-con-RNA-P3	hsa-miR-31-5p	15617308	17985385	10185	6839	652.16	380.253	-0.778268	4.40E-267	
RNA-con-RNA-P3	hsa-miR-455-5p	15617308	17985385	101	68	6.4672	3.7808	-0.77444969	0.0005457	
RNA-con-RNA-P3	hsa-miR-30e-3p	15617308	17985385	126	85	8.068	4.7261	-0.77156093	0.000118	
RNA-con-RNA-P3	hsa-miR-676-3p	15617308	17985385	37	25	2.3692	1.39	-0.76931511	0.0379276	
RNA-con-RNA-P3	hsa-miR-382-3p	15617308	17985385	1994	1348	127.68	74.9497	-0.76852552	1.04E-52	
RNA-con-RNA-P3	hsa-miR-411-5p	15617308	17985385	670	453	42.901	25.1871	-0.76832962	8.45E-19	
RNA-con-RNA-P3	hsa-miR-450a-5p	15617308	17985385	34	23	2.1771	1.2788	-0.76761703	0.0471124	

RNA-con-RNA-P3	hsa-miR-192-5p	15617308	17985385	4500	3059	288.14	170.083	-0.76054426	1.53E-114	
RNA-con-RNA-P3	hsa-miR-494	15617308	17985385	1274	869	81.576	48.317	-0.75561742	2.35E-33	
RNA-con-RNA-P3	hsa-miR-548k	15617308	17985385	41	28	2.6253	1.5568	-0.75389868	0.031728	
RNA-con-RNA-P3	hsa-miR-485-5p	15617308	17985385	2697	1861	172.69	103.473	-0.73895664	4.26E-66	
RNA-con-RNA-P3	hsa-miR-21-3p	15617308	17985385	1284	888	82.217	49.3734	-0.73569396	3.79E-32	
RNA-con-RNA-P3	hsa-let-7g-5p	15617308	17985385	1E+05	70201	6491.9	3903.22	-0.7339742	0	
RNA-con-RNA-P3	hsa-miR-9-3p	15617308	17985385	1855	1286	118.78	71.5025	-0.73220813	2.57E-45	
RNA-con-RNA-P3	hsa-miR-1291	15617308	17985385	156	109	9.9889	6.0605	-0.72088899	5.38E-05	
RNA-con-RNA-P3	hsa-miR-26a-5p	15617308	17985385	3924	2742	251.26	152.457	-0.72077595	2.53E-91	
RNA-con-RNA-P3	hsa-miR-543	15617308	17985385	2173	1531	139.14	85.1247	-0.7088927	5.77E-50	
RNA-con-RNA-P3	hsa-miR-183-5p	15617308	17985385	201	142	12.87	7.8953	-0.70497969	6.93E-06	
RNA-con-RNA-P3	hsa-miR-125b-5p	15617308	17985385	7010	4965	448.86	276.058	-0.70129995	4.34E-154	
RNA-con-RNA-P3	hsa-miR-1185-2-3p	15617308	17985385	48	34	3.0735	1.8904	-0.70119095	0.029059	
RNA-con-RNA-P3	hsa-miR-452-5p	15617308	17985385	1018	725	65.184	40.3105	-0.69336442	1.95E-23	
RNA-con-RNA-P3	hsa-miR-136-5p	15617308	17985385	392	281	25.1	15.6238	-0.68396497	9.66E-10	
RNA-con-RNA-P3	hsa-miR-107	15617308	17985385	1E+05	87320	7774.2	4855.05	-0.67920624	0	
RNA-con-RNA-P3	hsa-miR-16-2-3p	15617308	17985385	107	77	6.8514	4.2813	-0.67834988	0.0015278	
RNA-con-RNA-P3	hsa-miR-196a-5p	15617308	17985385	3835	2762	245.56	153.569	-0.67719291	3.42E-80	
RNA-con-RNA-P3	hsa-miR-10b-3p	15617308	17985385	18	13	1.1526	0.7228	-0.67322351	0.1987515	
RNA-con-RNA-P3	hsa-miR-1273c	15617308	17985385	18	13	1.1526	0.7228	-0.67322351	0.1987515	
RNA-con-RNA-P3	hsa-miR-497-5p	15617308	17985385	33	24	2.113	1.3344	-0.66310157	0.0851592	
RNA-con-RNA-P3	hsa-miR-382-5p	15617308	17985385	3743	2734	239.67	152.012	-0.65686127	2.73E-74	
RNA-con-RNA-P3	hsa-miR-500a-3p	15617308	17985385	82	60	5.2506	3.336	-0.654363	0.0072172	
RNA-con-RNA-P3	hsa-miR-378a-3p	15617308	17985385	2425	1782	155.28	99.0804	-0.64816698	1.14E-47	
RNA-con-RNA-P3	hsa-miR-191-5p	15617308	17985385	21717	16008	1390.6	890.056	-0.64371105	0	
RNA-con-RNA-P3	hsa-miR-1278	15617308	17985385	123	91	7.8759	5.0597	-0.63839295	0.0012801	
RNA-con-RNA-P3	hsa-miR-629-5p	15617308	17985385	104	77	6.6593	4.2813	-0.6373216	0.0031121	
RNA-con-RNA-P3	hsa-miR-21-5p	15617308	17985385	3E+05	2E+05	16763	10794.8	-0.634982	0	
RNA-con-RNA-P3	hsa-miR-182-5p	15617308	17985385	989	736	63.327	40.9221	-0.62994511	1.72E-19	
RNA-con-RNA-P3	hsa-miR-496	15617308	17985385	55	41	3.5217	2.2796	-0.62749132	0.0341039	
RNA-con-RNA-P3	hsa-miR-18a-5p	15617308	17985385	63	47	4.034	2.6132	-0.62639354	0.0235397	
RNA-con-RNA-P3	hsa-miR-3605-5p	15617308	17985385	40	30	2.5613	1.668	-0.61875695	0.0745067	
RNA-con-RNA-P3	hsa-miR-2355-3p	15617308	17985385	16	12	1.0245	0.6672	-0.61872879	0.2607179	
RNA-con-RNA-P3	hsa-miR-1255b-5p	15617308	17985385	20	15	1.2806	0.834	-0.61870063	0.2081575	
RNA-con-RNA-P3	hsa-miR-25-5p	15617308	17985385	11364	8548	727.65	475.275	-0.6144911	6.35E-197	
RNA-con-RNA-P3	hsa-miR-1262	15617308	17985385	122	92	7.8118	5.1153	-0.61083616	0.0020457	
RNA-con-RNA-P3	hsa-miR-574-5p	15617308	17985385	74	56	4.7383	3.1136	-0.60578593	0.0171877	
RNA-con-RNA-P3	hsa-miR-1271-5p	15617308	17985385	38	29	2.4332	1.6124	-0.59364522	0.093886	
RNA-con-RNA-P3	hsa-miR-374a-3p	15617308	17985385	581	444	37.202	24.6867	-0.59165782	6.06E-11	
RNA-con-RNA-P3	hsa-miR-484	15617308	17985385	47	36	3.0095	2.0016	-0.58837012	0.0644712	
RNA-con-RNA-P3	hsa-miR-125b-1-3p	15617308	17985385	60	46	3.8419	2.5576	-0.58702932	0.037032	

RNA-con-RNA-P3	hsa-miR-151a-3p	15617308	17985385	1055	810	67.553	45.0366	-0.58492833	2.64E-18	
RNA-con-RNA-P3	hsa-miR-181a-2-3p	15617308	17985385	1531	1176	98.032	65.3864	-0.58426658	8.52E-26	
RNA-con-RNA-P3	hsa-miR-424-3p	15617308	17985385	1058	814	67.745	45.259	-0.58191828	3.38E-18	
RNA-con-RNA-P3	hsa-miR-106b-5p	15617308	17985385	1275	982	81.64	54.5999	-0.58038141	1.64E-21	
RNA-con-RNA-P3	hsa-miR-320c	15617308	17985385	386	298	24.716	16.569	-0.57697042	1.86E-07	
RNA-con-RNA-P3	hsa-miR-30c-1-3p	15617308	17985385	31	24	1.985	1.3344	-0.57294781	0.1428399	
RNA-con-RNA-P3	hsa-miR-365a-5p	15617308	17985385	67	52	4.2901	2.8912	-0.56934287	0.0320122	
RNA-con-RNA-P3	hsa-miR-140-3p	15617308	17985385	1E+05	78363	6438	4357.04	-0.56327605	0	
RNA-con-RNA-P3	hsa-miR-548h-5p	15617308	17985385	689	543	44.118	30.1912	-0.54722948	3.15E-11	
RNA-con-RNA-P3	hsa-miR-194-5p	15617308	17985385	38	30	2.4332	1.668	-0.54473562	0.1209087	
RNA-con-RNA-P3	hsa-miR-93-5p	15617308	17985385	1648	1303	105.52	72.4477	-0.542558	2.22E-24	
RNA-con-RNA-P3	hsa-miR-425-3p	15617308	17985385	73	58	4.6743	3.2248	-0.53554064	0.0341715	
RNA-con-RNA-P3	hsa-miR-24-3p	15617308	17985385	13091	10436	838.24	580.249	-0.5306858	2.66E-174	
RNA-con-RNA-P3	hsa-miR-485-3p	15617308	17985385	576	460	36.882	25.5763	-0.52811714	4.16E-09	
RNA-con-RNA-P3	hsa-miR-106b-3p	15617308	17985385	684	547	43.798	30.4136	-0.52613522	1.73E-10	
RNA-con-RNA-P3	hsa-miR-125a-5p	15617308	17985385	1457	1174	93.294	65.2752	-0.51524778	6.22E-20	
RNA-con-RNA-P3	hsa-miR-708-5p	15617308	17985385	816	658	52.25	36.5853	-0.51415866	8.66E-12	
RNA-con-RNA-P3	hsa-miR-30e-5p	15617308	17985385	455	368	29.134	20.4611	-0.50983494	4.22E-07	
RNA-con-RNA-P3	hsa-miR-423-3p	15617308	17985385	1248	1016	79.911	56.4903	-0.50039636	1.77E-16	
RNA-con-RNA-P3	hsa-miR-125a-3p	15617308	17985385	140	114	8.9644	6.3385	-0.50006556	0.0058401	
RNA-con-RNA-P3	hsa-miR-103a-3p	15617308	17985385	2E+05	1E+05	10867	7743.62	-0.48881159	0	
RNA-con-RNA-P3	hsa-miR-493-5p	15617308	17985385	735	605	47.063	33.6384	-0.48449033	8.44E-10	
RNA-con-RNA-P3	hsa-miR-708-3p	15617308	17985385	176	145	11.27	8.0621	-0.48319592	0.0027414	
RNA-con-RNA-P3	hsa-miR-1185-1-3p	15617308	17985385	46	38	2.9455	2.1128	-0.47935635	0.1285536	
RNA-con-RNA-P3	hsa-miR-17-5p	15617308	17985385	614	510	39.315	28.3564	-0.47142015	4.51E-08	
RNA-con-RNA-P3	hsa-miR-361-5p	15617308	17985385	83	69	5.3146	3.8364	-0.47020796	0.0448482	
RNA-con-RNA-P3	hsa-miR-28-5p	15617308	17985385	149	124	9.5407	6.8945	-0.46864919	0.0073675	
RNA-con-RNA-P3	hsa-miR-767-5p	15617308	17985385	238	200	15.24	11.1201	-0.45464581	0.0009895	
RNA-con-RNA-P3	hsa-miR-345-5p	15617308	17985385	44	37	2.8174	2.0572	-0.45368235	0.1575005	
RNA-con-RNA-P3	hsa-miR-652-3p	15617308	17985385	20	17	1.2806	0.9452	-0.43812838	0.3556835	
RNA-con-RNA-P3	hsa-miR-9-5p	15617308	17985385	3967	3387	254.01	188.32	-0.43171917	1.31E-37	
RNA-con-RNA-P3	hsa-let-7i-5p	15617308	17985385	71777	61975	4596	3445.85	-0.41551452	0	
RNA-con-RNA-P3	hsa-miR-369-3p	15617308	17985385	276	240	17.673	13.3442	-0.40530965	0.0014285	
RNA-con-RNA-P3	hsa-miR-139-3p	15617308	17985385	56	49	3.5858	2.7244	-0.39635649	0.1591889	
RNA-con-RNA-P3	hsa-miR-410	15617308	17985385	88	77	5.6348	4.2813	-0.39631547	0.0777072	
RNA-con-RNA-P3	hsa-miR-30a-3p	15617308	17985385	2428	2130	155.47	118.43	-0.39259381	4.33E-20	
RNA-con-RNA-P3	hsa-miR-186-5p	15617308	17985385	346	304	22.155	16.9026	-0.39038063	0.0005657	
RNA-con-RNA-P3	hsa-miR-3613-3p	15617308	17985385	17	15	1.0885	0.834	-0.38422212	0.4497767	
RNA-con-RNA-P3	hsa-miR-28-3p	15617308	17985385	647	573	41.428	31.8592	-0.37891006	4.55E-06	
RNA-con-RNA-P3	hsa-miR-1304-5p	15617308	17985385	18	16	1.1526	0.8896	-0.37366323	0.4485843	
RNA-con-RNA-P3	hsa-miR-181a-3p	15617308	17985385	669	597	42.837	33.1936	-0.36795571	5.75E-06	

RNA-con-RNA-P3	hsa-miR-100-5p	15617308	17985385	7653	6855	490.03	381.143	-0.36254787	1.08E-51	
RNA-con-RNA-P3	hsa-miR-941	15617308	17985385	170	153	10.885	8.5069	-0.35568902	0.0267197	
RNA-con-RNA-P3	hsa-miR-329	15617308	17985385	349	315	22.347	17.5142	-0.35155608	0.0016952	
RNA-con-RNA-P3	hsa-miR-576-3p	15617308	17985385	21	19	1.3447	1.0564	-0.34812814	0.4434596	
RNA-con-RNA-P3	hsa-miR-125b-2-3p	15617308	17985385	128	116	8.196	6.4497	-0.34568793	0.0611768	
RNA-con-RNA-P3	hsa-miR-363-3p	15617308	17985385	97	88	6.2111	4.8929	-0.344159	0.1044951	
RNA-con-RNA-P3	hsa-miR-320d	15617308	17985385	44	40	2.8174	2.224	-0.34120762	0.2774696	
RNA-con-RNA-P3	hsa-miR-542-3p	15617308	17985385	23	21	1.4727	1.1676	-0.33491746	0.4391439	
RNA-con-RNA-P3	hsa-miR-148a-3p	15617308	17985385	461	421	29.519	23.4079	-0.3346239	0.0005737	
RNA-con-RNA-P3	hsa-miR-143-3p	15617308	17985385	1063	972	68.066	54.0439	-0.33279194	1.98E-07	
RNA-con-RNA-P3	hsa-miR-134	15617308	17985385	665	611	42.581	33.972	-0.32586368	5.50E-05	
RNA-con-RNA-P3	hsa-miR-486-5p	15617308	17985385	175	161	11.206	8.9517	-0.32397343	0.039498	
RNA-con-RNA-P3	hsa-miR-324-5p	15617308	17985385	107	99	6.8514	5.5045	-0.31578729	0.115857	
RNA-con-RNA-P3	hsa-miR-224-5p	15617308	17985385	175	162	11.206	9.0073	-0.31504041	0.0449254	
RNA-con-RNA-P3	hsa-miR-221-3p	15617308	17985385	85059	79673	5446.5	4429.87	-0.29805223	0	
RNA-con-RNA-P3	hsa-miR-378c	15617308	17985385	175	165	11.206	9.1741	-0.28856849	0.0649637	
RNA-con-RNA-P3	hsa-miR-369-5p	15617308	17985385	238	225	15.24	12.5102	-0.28471072	0.0336424	
RNA-con-RNA-P3	hsa-miR-376b-3p	15617308	17985385	137	130	8.7723	7.2281	-0.27933869	0.1132392	
RNA-con-RNA-P3	hsa-miR-20a-5p	15617308	17985385	214	204	13.703	11.3425	-0.27272153	0.0531386	
RNA-con-RNA-P3	hsa-miR-335-5p	15617308	17985385	573	548	36.69	30.4692	-0.26803922	0.0018672	
RNA-con-RNA-P3	hsa-miR-656	15617308	17985385	29	28	1.8569	1.5568	-0.25431251	0.5023144	
RNA-con-RNA-P3	hsa-miR-30a-5p	15617308	17985385	13642	13175	873.52	732.539	-0.25393147	4.62E-47	
RNA-con-RNA-P3	hsa-miR-3615	15617308	17985385	112	109	7.1715	6.0605	-0.24283808	0.2098947	
RNA-con-RNA-P3	hsa-miR-5481	15617308	17985385	18	18	1.1526	1.0008	-0.20373823	0.6656587	
RNA-con-RNA-P3	hsa-miR-337-5p	15617308	17985385	22	22	1.4087	1.2232	-0.20370409	0.634094	
RNA-con-RNA-P3	hsa-miR-1301	15617308	17985385	132	134	8.4522	7.4505	-0.18198966	0.3022439	
RNA-con-RNA-P3	hsa-miR-655	15617308	17985385	131	133	8.3881	7.3949	-0.18181342	0.304491	
RNA-con-RNA-P3	hsa-miR-15b-3p	15617308	17985385	100	102	6.4032	5.6713	-0.1751136	0.3863855	
RNA-con-RNA-P3	hsa-miR-30c-2-3p	15617308	17985385	1595	1648	102.13	91.63	-0.15651902	0.002014	
RNA-con-RNA-P3	hsa-miR-625-3p	15617308	17985385	58	60	3.7138	3.336	-0.15477683	0.5565079	
RNA-con-RNA-P3	hsa-miR-24-2-5p	15617308	17985385	110	115	7.0435	6.3941	-0.13955119	0.4658388	
RNA-con-RNA-P3	hsa-miR-148b-3p	15617308	17985385	226	237	14.471	13.1774	-0.13510885	0.3125606	
RNA-con-RNA-P3	hsa-miR-30b-3p	15617308	17985385	187	202	11.974	11.2313	-0.0923682	0.5258318	
RNA-con-RNA-P3	hsa-miR-15a-5p	15617308	17985385	1010	1113	64.672	61.8836	-0.06357964	0.3098802	
RNA-con-RNA-P3	hsa-miR-193b-5p	15617308	17985385	175	194	11.206	10.7865	-0.05498021	0.7115965	
RNA-con-RNA-P3	hsa-miR-503-5p	15617308	17985385	585	653	37.458	36.3073	-0.04502963	0.5819333	
RNA-con-RNA-P3	hsa-miR-2110	15617308	17985385	137	155	8.7723	8.6181	-0.02558531	0.8757049	
RNA-con-RNA-P3	hsa-miR-598	15617308	17985385	1072	1226	68.642	68.1665	-0.01002448	0.8665113	
RNA-con-RNA-P3	hsa-miR-342-5p	15617308	17985385	19	22	1.2166	1.2232	0.0078054	0.9979859	
RNA-con-RNA-P3	hsa-miR-30b-5p	15617308	17985385	55	64	3.5217	3.5584	0.01495668	0.9620919	
RNA-con-RNA-P3	hsa-miR-1266	15617308	17985385	36	42	2.3051	2.3352	0.01871678	0.963241	

RNA-con-RNA-P3	hsa-miR-376c-3p	15617308	17985385	702	820	44.95	45.5926	0.02047536	0.7844566	
RNA-con-RNA-P3	hsa-miR-2682-5p	15617308	17985385	17	20	1.0885	1.112	0.03081538	0.9614002	
RNA-con-RNA-P3	hsa-miR-92b-3p	15617308	17985385	1966	2320	125.89	128.994	0.03518164	0.4270539	
RNA-con-RNA-P3	hsa-miR-29a-3p	15617308	17985385	2E+05	2E+05	10529	10890.1	0.04864963	6.34E-24	
RNA-con-RNA-P3	hsa-miR-374b-5p	15617308	17985385	544	648	34.833	36.0293	0.04871175	0.5633631	
RNA-con-RNA-P3	hsa-miR-138-5p	15617308	17985385	1866	2229	119.48	123.934	0.05276908	0.2441612	
RNA-con-RNA-P3	hsa-miR-222-5p	15617308	17985385	59	71	3.7779	3.9476	0.0633913	0.8100531	
RNA-con-RNA-P3	hsa-miR-138-1-3p	15617308	17985385	18	22	1.1526	1.2232	0.08576839	0.8650759	
RNA-con-RNA-P3	hsa-miR-210	15617308	17985385	603	740	38.611	41.1445	0.09168767	0.247468	
RNA-con-RNA-P3	hsa-miR-29c-3p	15617308	17985385	878	1086	56.22	60.3824	0.10305235	0.1156935	
RNA-con-RNA-P3	hsa-miR-574-3p	15617308	17985385	59	73	3.7779	4.0589	0.10350428	0.6889447	
RNA-con-RNA-P3	hsa-miR-421	15617308	17985385	74	93	4.7383	5.1709	0.12604586	0.580696	
RNA-con-RNA-P3	hsa-miR-339-5p	15617308	17985385	167	212	10.693	11.7873	0.14052615	0.3488408	
RNA-con-RNA-P3	hsa-miR-10b-5p	15617308	17985385	1428	1818	91.437	101.082	0.14467757	0.0045258	
RNA-con-RNA-P3	hsa-miR-181b-5p	15617308	17985385	10719	13741	686.35	764.009	0.15463736	7.87E-17	
RNA-con-RNA-P3	hsa-miR-625-5p	15617308	17985385	35	45	2.2411	2.502	0.15887476	0.6343452	
RNA-con-RNA-P3	hsa-miR-96-5p	15617308	17985385	124	160	7.9399	8.8961	0.16405217	0.3447056	
RNA-con-RNA-P3	hsa-miR-136-3p	15617308	17985385	23	30	1.4727	1.668	0.17965572	0.6653017	
RNA-con-RNA-P3	hsa-miR-197-5p	15617308	17985385	16	21	1.0245	1.1676	0.18862613	0.7087368	
RNA-con-RNA-P3	hsa-miR-146b-5p	15617308	17985385	676	888	43.285	49.3734	0.18985684	0.0098346	
RNA-con-RNA-P3	hsa-miR-10a-3p	15617308	17985385	19	25	1.2166	1.39	0.19222997	0.675114	
RNA-con-RNA-P3	hsa-miR-377-3p	15617308	17985385	44	59	2.8174	3.2804	0.21950734	0.4515775	
RNA-con-RNA-P3	hsa-miR-409-5p	15617308	17985385	187	252	11.974	14.0114	0.22670799	0.1037574	
RNA-con-RNA-P3	hsa-miR-22-5p	15617308	17985385	474	639	30.351	35.5288	0.22724966	0.0092385	
RNA-con-RNA-P3	hsa-miR-1294	15617308	17985385	17	23	1.0885	1.2788	0.23244924	0.6288371	
RNA-con-RNA-P3	hsa-miR-193a-5p	15617308	17985385	89	121	5.6988	6.7277	0.23945521	0.2367812	
RNA-con-RNA-P3	hsa-miR-27a-3p	15617308	17985385	5822	8022	372.79	446.029	0.2587679	1.33E-25	
RNA-con-RNA-P3	hsa-let-7e-3p	15617308	17985385	13	18	0.8324	1.0008	0.26580482	0.6296867	
RNA-con-RNA-P3	hsa-miR-487a	15617308	17985385	18	25	1.1526	1.39	0.27019296	0.5577633	
RNA-con-RNA-P3	hsa-miR-135b-5p	15617308	17985385	126	175	8.068	9.7301	0.27024355	0.1092122	
RNA-con-RNA-P3	hsa-miR-548a-3p	15617308	17985385	23	32	1.4727	1.7792	0.27276512	0.4998755	
RNA-con-RNA-P3	hsa-miR-4488	15617308	17985385	553	772	35.409	42.9237	0.27764204	0.0005282	
RNA-con-RNA-P3	hsa-miR-23b-3p	15617308	17985385	2436	3402	155.98	189.154	0.27818978	2.96E-13	
RNA-con-RNA-P3	hsa-miR-154-3p	15617308	17985385	110	154	7.0435	8.5625	0.28173958	0.1182024	
RNA-con-RNA-P3	hsa-miR-455-3p	15617308	17985385	146	205	9.3486	11.3981	0.28597112	0.0670929	
RNA-con-RNA-P3	hsa-miR-34c-5p	15617308	17985385	151	213	9.6688	11.8429	0.29261365	0.0563914	
RNA-con-RNA-P3	hsa-miR-365b-5p	15617308	17985385	51	72	3.2656	4.0033	0.29384165	0.2693873	
RNA-con-RNA-P3	hsa-miR-23a-5p	15617308	17985385	84	120	5.3786	6.6721	0.31091021	0.1305338	
RNA-con-RNA-P3	hsa-miR-151b	15617308	17985385	14	20	0.8964	1.112	0.31094223	0.5517328	
RNA-con-RNA-P3	hsa-miR-362-3p	15617308	17985385	34	49	2.1771	2.7244	0.32353086	0.3206376	
RNA-con-RNA-P3	hsa-miR-424-5p	15617308	17985385	257	374	16.456	20.7947	0.33759341	0.0037396	

RNA-con-RNA-P3	hsa-miR-589-3p	15617308	17985385	30	44	1.9209	2.4464	0.3488779	0.3130569	
RNA-con-RNA-P3	hsa-miR-376a-3p	15617308	17985385	121	178	7.7478	9.8969	0.35318999	0.03735	
RNA-con-RNA-P3	hsa-miR-942	15617308	17985385	38	56	2.4332	3.1136	0.35572871	0.2444301	
RNA-con-RNA-P3	hsa-miR-92a-3p	15617308	17985385	2520	3729	161.36	207.335	0.36168605	1.29E-22	
RNA-con-RNA-P3	hsa-let-7d-3p	15617308	17985385	73	109	4.6743	6.0605	0.37468649	0.0859699	
RNA-con-RNA-P3	hsa-miR-10a-5p	15617308	17985385	2681	4020	171.67	223.515	0.38074502	1.56E-26	
RNA-con-RNA-P3	hsa-miR-195-5p	15617308	17985385	21	33	1.3447	1.8348	0.44833847	0.2717028	
RNA-con-RNA-P3	hsa-miR-129-5p	15617308	17985385	84	133	5.3786	7.3949	0.45929994	0.0217644	
RNA-con-RNA-P3	hsa-miR-122-5p	15617308	17985385	48	76	3.0735	4.2257	0.45930786	0.0841209	
RNA-con-RNA-P3	hsa-miR-193b-3p	15617308	17985385	32	51	2.049	2.8356	0.46873405	0.1512934	
RNA-con-RNA-P3	hsa-miR-27a-5p	15617308	17985385	32	51	2.049	2.8356	0.46873405	0.1512934	
RNA-con-RNA-P3	hsa-miR-582-3p	15617308	17985385	13	21	0.8324	1.1676	0.48819725	0.3488625	
RNA-con-RNA-P3	hsa-miR-423-5p	15617308	17985385	7905	12894	506.17	716.915	0.50218316	3.75E-134	
RNA-con-RNA-P3	hsa-miR-532-3p	15617308	17985385	76	125	4.8664	6.9501	0.51417883	0.0136714	
RNA-con-RNA-P3	hsa-miR-1293	15617308	17985385	18	30	1.1526	1.668	0.53322736	0.2199173	
RNA-con-RNA-P3	hsa-miR-16-5p	15617308	17985385	4761	8003	304.85	444.972	0.5455969	2.52E-97	
RNA-con-RNA-P3	hsa-miR-760	15617308	17985385	44	75	2.8174	4.1701	0.56571758	0.0379874	
RNA-con-RNA-P3	hsa-miR-541-5p	15617308	17985385	41	70	2.6253	3.892	0.56802942	0.0443461	
RNA-con-RNA-P3	hsa-miR-4707-3p	15617308	17985385	14	24	0.8964	1.3344	0.57397664	0.2435038	
RNA-con-RNA-P3	hsa-miR-3934-5p	15617308	17985385	18	31	1.1526	1.7236	0.58053308	0.177641	
RNA-con-RNA-P3	hsa-miR-23a-3p	15617308	17985385	19319	33292	1237	1851.06	0.58147583	0	
RNA-con-RNA-P3	hsa-miR-1224-5p	15617308	17985385	11	19	0.7043	1.0564	0.58489422	0.2948036	
RNA-con-RNA-P3	hsa-miR-548t-5p	15617308	17985385	20	35	1.2806	1.946	0.6036918	0.1369376	
RNA-con-RNA-P3	hsa-miR-29b-3p	15617308	17985385	1906	3348	122.04	186.151	0.60907159	1.66E-50	
RNA-con-RNA-P3	hsa-miR-181d	15617308	17985385	1373	2421	87.915	134.609	0.61459192	1.42E-37	
RNA-con-RNA-P3	hsa-miR-34a-5p	15617308	17985385	17	30	1.0885	1.668	0.61577788	0.1623692	
RNA-con-RNA-P3	hsa-miR-145-5p	15617308	17985385	70	125	4.4822	6.9501	0.63282671	0.0029534	
RNA-con-RNA-P3	hsa-miR-361-3p	15617308	17985385	14	25	0.8964	1.39	0.63287033	0.1931903	
RNA-con-RNA-P3	hsa-miR-331-3p	15617308	17985385	202	368	12.934	20.4611	0.66167058	1.01E-07	
RNA-con-RNA-P3	hsa-miR-30c-5p	15617308	17985385	227	415	14.535	23.0743	0.66673596	1.24E-08	
RNA-con-RNA-P3	hsa-miR-154-5p	15617308	17985385	70	130	4.4822	7.2281	0.68940944	0.0010735	
RNA-con-RNA-P3	hsa-miR-449c-5p	15617308	17985385	45	84	2.8814	4.6705	0.69680705	0.0081371	
RNA-con-RNA-P3	hsa-miR-218-1-3p	15617308	17985385	16	30	1.0245	1.668	0.7031993	0.1159359	
RNA-con-RNA-P3	hsa-miR-495-3p	15617308	17985385	680	1289	43.541	71.6693	0.71896747	7.14E-27	
RNA-con-RNA-P3	hsa-miR-181c-5p	15617308	17985385	69	131	4.4182	7.2837	0.72121277	0.0006365	
RNA-con-RNA-P3	hsa-miR-193a-3p	15617308	17985385	120	228	7.6838	12.677	0.7223215	6.14E-06	
RNA-con-RNA-P3	hsa-miR-885-3p	15617308	17985385	42	80	2.6893	4.4481	0.72595852	0.0074542	
RNA-con-RNA-P3	hsa-miR-641	15617308	17985385	16	31	1.0245	1.7236	0.75050502	0.0904725	
RNA-con-RNA-P3	hsa-miR-181a-5p	15617308	17985385	5670	11004	363.06	611.83	0.75292826	6.17E-234	
RNA-con-RNA-P3	hsa-miR-7-1-3p	15617308	17985385	17	33	1.0885	1.8348	0.75328141	0.0793586	
RNA-con-RNA-P3	hsa-miR-4449	15617308	17985385	35	68	2.2411	3.7808	0.75448451	0.0108453	

RNA-con-RNA-P3	hsa-miR-411-3p	15617308	17985385	70	137	4.4822	7.6173	0.76507269	0.0002355	
RNA-con-RNA-P3	hsa-miR-431-3p	15617308	17985385	28	55	1.7929	3.058	0.77029338	0.0199352	
RNA-con-RNA-P3	hsa-miR-615-5p	15617308	17985385	51	101	3.2656	5.6157	0.78211778	0.0013141	
RNA-con-RNA-P3	hsa-miR-222-3p	15617308	17985385	16946	33798	1085.1	1879.19	0.79231384	0	
RNA-con-RNA-P3	hsa-miR-100-3p	15617308	17985385	14	28	0.8964	1.5568	0.79636906	0.0910527	
RNA-con-RNA-P3	hsa-miR-381-5p	15617308	17985385	10	21	0.6403	1.1676	0.8667262	0.1183745	
RNA-con-RNA-P3	hsa-miR-1180	15617308	17985385	9	19	0.5763	1.0564	0.87426428	0.1357661	
RNA-con-RNA-P3	hsa-miR-1307-3p	15617308	17985385	681	1446	43.606	80.3986	0.88266026	7.07E-42	
RNA-con-RNA-P3	hsa-miR-324-3p	15617308	17985385	15	32	0.9605	1.7792	0.88937117	0.0464857	
RNA-con-RNA-P3	hsa-miR-22-3p	15617308	17985385	1972	4250	126.27	236.303	0.90412374	1.93E-124	
RNA-con-RNA-P3	hsa-miR-92b-5p	15617308	17985385	297	651	19.017	36.1961	0.92851424	2.37E-21	
RNA-con-RNA-P3	hsa-miR-337-3p	15617308	17985385	17	38	1.0885	2.1128	0.9568148	0.0206863	
RNA-con-RNA-P3	hsa-miR-29a-5p	15617308	17985385	59	133	3.7779	7.3949	0.96894612	9.58E-06	
RNA-con-RNA-P3	hsa-miR-370	15617308	17985385	10	23	0.6403	1.2788	0.99797073	0.0652006	
RNA-con-RNA-P3	hsa-miR-769-3p	15617308	17985385	10	23	0.6403	1.2788	0.99797073	0.0652006	
RNA-con-RNA-P3	hsa-miR-31-3p	15617308	17985385	55	128	3.5217	7.1169	1.01497695	6.45E-06	**
RNA-con-RNA-P3	hsa-miR-505-3p	15617308	17985385	11	26	0.7043	1.4456	1.03740642	0.0423474	*
RNA-con-RNA-P3	hsa-miR-582-5p	15617308	17985385	11	27	0.7043	1.5012	1.09185421	0.0308942	*
RNA-con-RNA-P3	hsa-miR-363-5p	15617308	17985385	7	18	0.4482	1.0008	1.15893914	0.0676627	
RNA-con-RNA-P3	hsa-miR-197-3p	15617308	17985385	17	44	1.0885	2.4464	1.1683189	0.0033337	**
RNA-con-RNA-P3	hsa-miR-19b-3p	15617308	17985385	118	309	7.5557	17.1806	1.1851431	1.65E-15	**
RNA-con-RNA-P3	hsa-miR-15b-5p	15617308	17985385	900	2365	57.628	131.496	1.19016375	1.08E-108	**
RNA-con-RNA-P3	hsa-miR-29c-5p	15617308	17985385	9	24	0.5763	1.3344	1.21129927	0.0275178	*
RNA-con-RNA-P3	hsa-miR-146a-5p	15617308	17985385	18	51	1.1526	2.8356	1.29876211	0.0005825	**
RNA-con-RNA-P3	hsa-miR-1306-5p	15617308	17985385	18	52	1.1526	2.8912	1.32677649	0.0004134	**
RNA-con-RNA-P3	hsa-miR-539-5p	15617308	17985385	12	35	0.7684	1.946	1.34058229	0.003698	**
RNA-con-RNA-P3	hsa-miR-92a-1-5p	15617308	17985385	334	1019	21.387	56.6571	1.40555636	2.43E-61	**
RNA-con-RNA-P3	hsa-miR-365a-3p	15617308	17985385	19	59	1.2166	3.2804	1.43101683	6.64E-05	**
RNA-con-RNA-P3	hsa-miR-365b-3p	15617308	17985385	19	59	1.2166	3.2804	1.43101683	6.64E-05	**
RNA-con-RNA-P3	hsa-miR-425-5p	15617308	17985385	20	64	1.2806	3.5584	1.47440878	2.13E-05	**
RNA-con-RNA-P3	hsa-miR-4508	15617308	17985385	41	132	2.6253	7.3393	1.48316018	7.08E-10	**
RNA-con-RNA-P3	hsa-miR-551b-3p	15617308	17985385	13	42	0.8324	2.3352	1.48819725	0.0005622	**
RNA-con-RNA-P3	hsa-miR-221-5p	15617308	17985385	6164	22227	394.69	1235.84	1.64669527	0	**
RNA-con-RNA-P3	hsa-miR-4521	15617308	17985385	13	47	0.8324	2.6132	1.65046867	8.20E-05	**
RNA-con-RNA-P3	hsa-miR-129-2-3p	15617308	17985385	25	92	1.6008	5.1153	1.67602578	2.03E-08	**
RNA-con-RNA-P3	hsa-miR-1246	15617308	17985385	34	126	2.1771	7.0057	1.68612153	3.97E-11	**
RNA-con-RNA-P3	hsa-miR-342-3p	15617308	17985385	214	813	13.703	45.2034	1.7219711	1.38E-65	**
RNA-con-RNA-P3	hsa-miR-550a-3p	15617308	17985385	6	24	0.3842	1.3344	1.79626177	0.0032354	**
RNA-con-RNA-P3	hsa-miR-7-5p	15617308	17985385	46	185	2.9455	10.2861	1.80411163	4.36E-17	**
RNA-con-RNA-P3	hsa-miR-935	15617308	17985385	15	63	0.9605	3.5028	1.8666511	6.28E-07	**
RNA-con-RNA-P3	hsa-miR-299-5p	15617308	17985385	92	395	5.8909	21.9623	1.89846918	3.82E-37	**

RNA-con-RNA-P3	hsa-miR-132-3p	15617308	17985385	6	26	0.3842	1.4456	1.91173899	0.0013642	**
RNA-con-RNA-P3	hsa-miR-487b	15617308	17985385	337	1467	21.579	81.5662	1.91837017	3.58E-135	**
RNA-con-RNA-P3	hsa-miR-129-1-3p	15617308	17985385	7	31	0.4482	1.7236	1.94321045	0.0003865	**
RNA-con-RNA-P3	hsa-miR-4454	15617308	17985385	7	33	0.4482	1.8348	2.03340826	0.0001592	**
RNA-con-RNA-P3	hsa-miR-335-3p	15617308	17985385	8	39	0.5123	2.1684	2.08157012	2.91E-05	**
RNA-con-RNA-P3	hsa-miR-3676-3p	15617308	17985385	4	21	0.2561	1.1676	2.18876696	0.0018331	**
RNA-con-RNA-P3	hsa-miR-155-5p	15617308	17985385	249	1308	15.944	72.7257	2.18946976	8.87E-143	**
RNA-con-RNA-P3	hsa-miR-29b-1-5p	15617308	17985385	22	120	1.4087	6.6721	2.24377651	7.26E-15	**
RNA-con-RNA-P3	hsa-miR-4657	15617308	17985385	6	38	0.3842	2.1128	2.45922678	5.10E-06	**
RNA-con-RNA-P3	hsa-miR-576-5p	15617308	17985385	5	34	0.3202	1.8904	2.56164632	1.06E-05	**
RNA-con-RNA-P3	hsa-miR-431-5p	15617308	17985385	11	75	0.7043	4.1701	2.56581999	3.42E-11	**
RNA-con-RNA-P3	hsa-miR-3654	15617308	17985385	6	43	0.3842	2.3908	2.63756402	4.28E-07	**
RNA-con-RNA-P3	hsa-let-7b-3p	15617308	17985385	10	79	0.6403	4.3925	2.77822237	1.20E-12	**
RNA-con-RNA-P3	hsa-miR-18a-3p	15617308	17985385	4	34	0.2561	1.8904	2.88391238	2.76E-06	**
RNA-con-RNA-P3	hsa-miR-5096	15617308	17985385	2	18	0.1281	1.0008	2.96581131	0.0007077	**
RNA-con-RNA-P3	hsa-miR-362-5p	15617308	17985385	5	45	0.3202	2.502	2.96603658	3.77E-08	**
RNA-con-RNA-P3	hsa-miR-204-5p	15617308	17985385	16	164	1.0245	9.1185	3.15387654	5.54E-28	**
RNA-con-RNA-P3	hsa-miR-6087	15617308	17985385	21	217	1.3447	12.0654	3.16551949	1.07E-36	**
RNA-con-RNA-P3	hsa-miR-664a-3p	15617308	17985385	2	33	0.1281	1.8348	3.84028043	1.71E-07	**
RNA-con-RNA-P3	hsa-miR-3614-3p	15617308	17985385	1	19	0.064	1.0564	4.04494049	7.67E-05	**
RNA-con-RNA-P3	hsa-miR-887	15617308	17985385	4	121	0.2561	6.7277	4.71533422	7.40E-28	**
RNA-con-RNA-P3	hsa-miR-3674	15617308	17985385	1	80	0.064	4.4481	6.11897351	7.95E-21	**
RNA-con-RNA-P3	hsa-miR-3614-5p	15617308	17985385	1	99	0.064	5.5045	6.42639581	6.79E-26	**
RNA-con-RNA-P3	hsa-miR-155-3p	15617308	17985385	0	31	0.01	1.7236	7.42928119	4.12E-09	**
RNA-con-RNA-P3	hsa-miR-206	15617308	17985385	12	3268	0.7684	181.703	7.8855098	0	**
RNA-con-RNA-P3	hsa-miR-3687	15617308	17985385	2	764	0.1281	42.4789	8.37333212	2.77E-203	**
RNA-con-RNA-P3	hsa-miR-3648	15617308	17985385	2	817	0.1281	45.4258	8.47009764	1.30E-217	**
RNA-con-RNA-P3	hsa-miR-663a	15617308	17985385	0	124	0.01	6.8945	9.42930212	2.34E-34	**

Table S1. MiRNA profile results. Human astrocytoma cells (U251) were mock infected or infected with JEV P3 (MOI = 5) for 36h. The cellular total RNAs were extracted using TRIzol Reagent (Invitrogen), and subjected to high throughput sequencing by IIlumina Genome Analyzer.
